# Farnesoid X receptor mediates macrophage-intrinsic responses to suppress colitis-induced colon cancer progression

**DOI:** 10.1172/jci.insight.170428

**Published:** 2024-01-23

**Authors:** Xingchen Dong, Ming Qi, Chunmiao Cai, Yu Zhu, Yuwenbin Li, Sally Coulter, Fei Sun, Christopher Liddle, Nataliya V. Uboha, Richard Halberg, Wei Xu, Paul Marker, Ting Fu

**Affiliations:** 1 Pharmaceutical Sciences Division, School of Pharmacy, University of Wisconsin Carbone Cancer Center (UWCCC), University of Wisconsin–Madison, Madison, Wisconsin, USA.; 2 Department of Pathology, School of Medicine, Stanford University, Palo Alto, California, USA.; 3 Gene Expression Laboratory, Salk Institute for Biological Studies, La Jolla, California, USA.; 4 Storr Liver Centre, The Westmead Institute for Medical Research and Sydney Medical School, University of Sydney, Westmead Hospital, Westmead, New South Wales, Australia.; 5 Department of Medicine, UWCCC, and; 6 McArdle Laboratory for Cancer Research, School of Medicine and Public Health, University of Wisconsin–Madison, Madison, Wisconsin, USA.

**Keywords:** Endocrinology, Gastroenterology, Colorectal cancer

## Abstract

Bile acids (BAs) affect the intestinal environment by ensuring barrier integrity, maintaining microbiota balance, regulating epithelium turnover, and modulating the immune system. As a master regulator of BA homeostasis, farnesoid X receptor (FXR) is severely compromised in patients with inflammatory bowel disease (IBD) and colitis-associated colorectal cancer (CAC). At the front line, gut macrophages react to the microbiota and metabolites that breach the epithelium. We aim to study the role of the BA/FXR axis in macrophages. This study demonstrates that inflammation-induced epithelial abnormalities compromised FXR signaling and altered BAs’ profile in a mouse CAC model. Further, gut macrophage–intrinsic FXR sensed aberrant BAs, leading to pro-inflammatory cytokines’ secretion, which promoted intestinal stem cell proliferation. Mechanistically, activation of FXR ameliorated intestinal inflammation and inhibited colitis-associated tumor growth, by regulating gut macrophages’ recruitment, polarization, and crosstalk with Th17 cells. However, deletion of FXR in bone marrow or gut macrophages escalated the intestinal inflammation. In summary, our study reveals a distinctive regulatory role of FXR in gut macrophages, suggesting its potential as a therapeutic target for addressing IBD and CAC.

## Abstract 2

<p>Farnesoid X receptor modulates gut macrophages in response to aberrant bile acids in a colitis-associated colon cancer model.</p>

## Graphical abstract

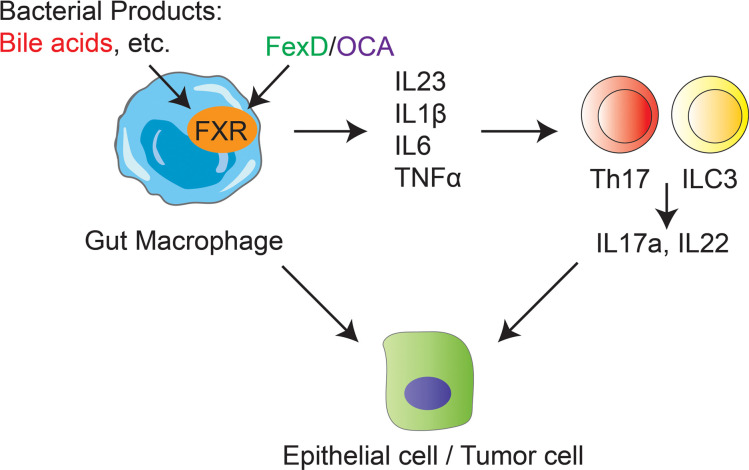

## Introduction

Environmental risk factors of colorectal cancer (CRC) include diet, carcinogenic metabolites, gut microbes, and intestinal inflammation (1, 2). Colitis-associated cancer (CAC) is one subtype of CRC associated with inflammatory bowel disease (IBD), a chronic intestinal disorder that affects 4 million individuals worldwide (3, 4). Among IBD patients, more than 20% develop CAC, and more than 50% die of CAC. Compared with sporadic CRC, CAC has higher malignancy, implying that inflammation facilitates tumor progression (3, 4). While the underlying causes are not fully understood, impaired epithelial barrier function and aberrant immune responses are 2 leading causes (2, 5–7). Increased levels of the cytokines, like TNF-α, IL-17A, and IL-23, have been associated with the pathogenesis in patients with and mouse models of CRC (8–15). Notably, the macrophage is a primary source for IL-23 and IL-1β (16, 17).

Loss of tight epithelial junctions exposes macrophages to a pathophysiological concentration of metabolites like bile acids (BAs), thus inducing cytokine production and imbalanced gut microbiota (5, 7, 18). BAs are cholesterol-derived metabolites that facilitate lipid absorption and mediate postprandial response (19). As early and dynamic sensors of dietary and microbiome status, BAs modulate gut physiology and metabolism, partly through affecting the activity of their receptor, farnesoid X receptor (FXR) (2, 20, 21). Reversely, both synthesis and transport of BAs are tightly controlled by FXR (19). The loss of BAs’ homeostasis has been associated with the pathogenesis of obesity, IBD, and CRC (2, 21–24). Recent studies also show a couple of microbial BAs can modulate the function of effector T cells, including Th17 and Treg cells (25–28). However, whether BAs directly modulate gut macrophages’ function remains inconclusive.

Located strategically in the subepithelial lamina propria, close to luminal dietary and microbial stimuli, gut macrophages mediate inflammatory responses to food, bacteria, and metabolites like BAs that breach the epithelium (29). Gut macrophages not only protect against harmful microbes and scavenge dead cells but also activate adaptive immune cells by eliciting cytokines and presenting antigens (30, 31). For example, IL-23 produced by macrophages promotes Th17 cell and innate lymphoid cell 3 maturation and activation (8, 17, 32). Moreover, the dynamic changes of macrophage polarization and inflammation-elicited cytokines’ production emphasize the importance of investigating how BAs reshape the gut macrophages at the early phase of CAC development.

## Results

### Inflammation disrupts BAs’ homeostasis in CAC mouse model.

**Figure 1:**
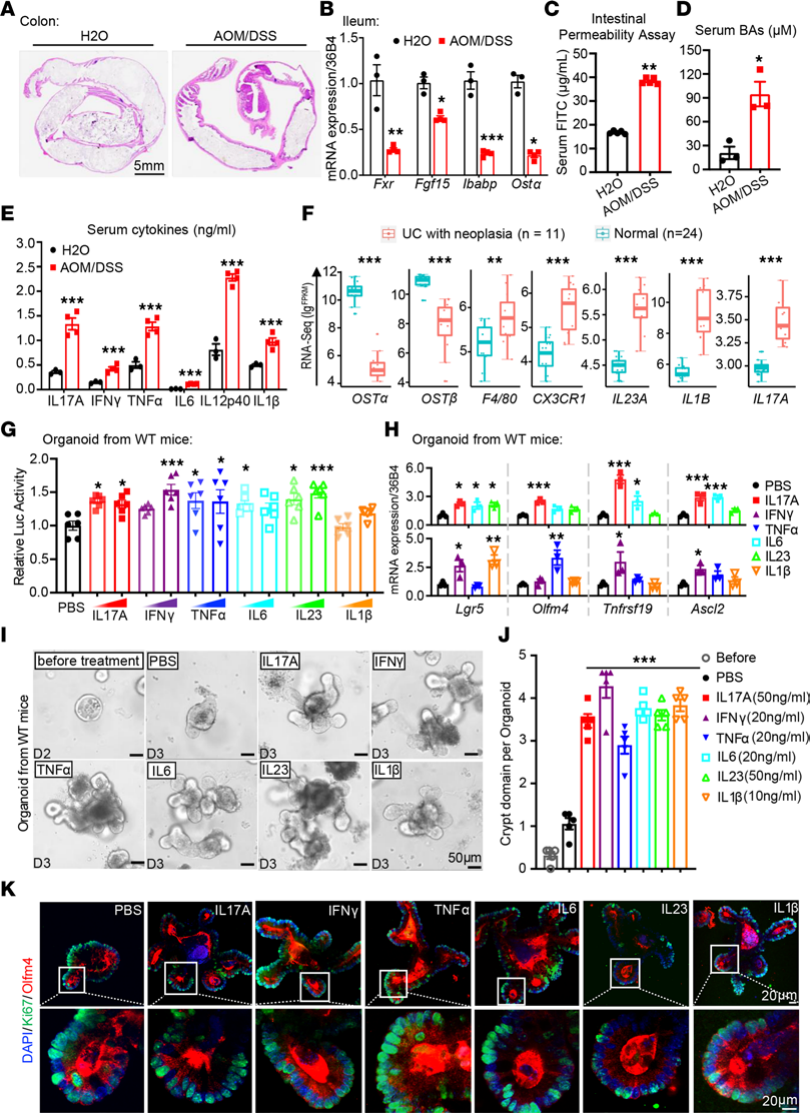
Cytokines increased in CAC model stimulate ISCs’ proliferation. (**A**) H&E staining of colon parts, scale bar 5 mm. (**B**) Expression of FXR and its downstream targets (*Fgf15*, *Ibabp*, *Ost*α) is reduced in CAC mice. (**C**–**E**) Intestinal permeability (**C**), total serum BAs (**D**), and serum cytokine levels (**E**) in CAC mice. (**F**) Relative expression (fragments per kilobase million [FPKM] values) of presented genes based on RNA-Seq data of healthy and CAC patients (Supplemental Table 3). Box plots show the interquartile range (box), median (line), and minimum and maximum (whiskers). (**G**) Proliferation of intestinal organoids from WT mice, measured by ATP luminescence, in response to increasing concentrations of IL17A (50, 100 ng/mL), IFN-γ (10, 20 ng/mL), TNF-α (20, 40 ng/mL), IL6 (20, 50 ng/mL), IL23 (50, 100 ng/mL), and IL1β (10, 20 ng/mL). (**H**) Stem cell marker (*Lgr5*, *Olfm4*, *Tnfrsf19*, *Ascl2*) genes’ expression in WT organoids treated with vehicle (PBS) and cytokines of indicated concentration. Experiments in **H**–**K** are conducted under same conditions as in **H**. (**I** and **J**) Bright-field images of branching WT organoids for 24 hours of treatment (**I**). Scale bar 50 μm. Branching was quantified as crypt domain per organoid from 5 individuals (**J**). (**K**) Images of WT organoids treated with different cytokines, co-immunostained with stem cell marker Olfm4 (red) and proliferating marker Ki67 (green); the nucleus is counterstained with DAPI (blue). Circled parts with higher magnification are presented (bottom). Scale bar 20 μm. *n* = 3–5/group. Experiments were independently replicated twice, and representative data are shown as mean ± SEM. *P* values determined with Student’s unpaired *t* test (**B**–**E**), Wilcoxon test (**F**), and 1-way ANOVA test followed by Tukey’s multiple comparisons (**G**, **H**, and **J**). *P < 0.05; **P < 0.01; ***P < 0.005.

The crypt-villus architecture is fundamental for intestine regeneration and BAs’ homeostasis. Both inflammation and tumor initiation could disrupt the bottom-to-top crypt-villus structure. We employed the azoxymethane (AOM)/dextran sodium sulfate (DSS) mouse model of CAC (3, 33) to explore the consequences of chronic inflammation on intestinal stem cell (ISC) proliferation (Supplemental Figure 1A; supplemental material available online with this article; https://doi.org/10.1172/jci.insight.170428DS1) (23). AOM/DSS mice developed hyperplasia in both small intestine and colon, with only multiple adenomas and adenocarcinomas in the colon (Figure 1A). These changes were associated with reduced expression of *Fxr* and its target genes in intestinal epithelial cells (IECs) and increased intestinal permeability (Figure 1, B and C) (18, 34). Consistent with reduced FXR signaling, total BA levels were also increased, with a disproportionate increase in ex vivo direct effect of FXR (Figure 1D and Supplemental Figure 1, B and C). Furthermore, pro-inflammatory cytokines such as IL17A and IL1β dramatically increased in the serum (Figure 1E). In addition, we observed marked increases in cytokines such as G-CSF and GM-CSF (Supplemental Figure 1D). To determine if the AOM/DSS mouse model mimics human at the transcriptional level, we analyzed the gene expression profile of patients with CRC and CAC (Figure 1F and Supplemental Figure 1, E and F) (35). Consistently, the expression of *FXR* and its target genes was profoundly decreased in tumors compared with healthy tissues, together with markedly increased expression of pro-inflammatory cytokines such as *IL17A* and *IL23* (Figure 1F). Taken together, these data illustrated a disrupted FXR signaling accompanied by exacerbated inflammation during CAC progression.

### Cytokines increased in CAC stimulate intestinal stem cells’ proliferation.

Cancer stem cells (CSCs) are the driving force for tumor growth and, similar to ISCs (Lgr5^+^), fuel normal epithelium turnover (6, 24, 36). Moreover, mice and humans’ CSCs are capable of tumor initiation and giving rise to a highly proliferative tumor (6, 36). Inflammation resulting from epithelium injury alters the plasticity of ISCs (3). To investigate the impact of pro-inflammatory cytokines on the proliferation and stemness of ISCs, we treated intestinal organoids generated from WT mice with different cytokines. Compared with PBS control, pro-inflammatory cytokines such as IL17A and IL23 profoundly induced organoid growth (Figure 1G and Supplemental Figure 1, G and H). Likewise, the expression of ISC signature genes (*Lgr5*, *Olfm4*, etc.) and CSC marker gene (*Ascl2*) was substantially upregulated (Figure 1H). Of note, pro-inflammatory cytokines also upregulated expression of Paneth cell and goblet cell markers, which is in accordance with the overall organoid growth (Supplemental Figure 1H). Additionally, cytokine-induced organoids’ growth was demonstrated by organoid budding and branching (Figure 1, I and J, and Supplemental Figure 1I), as well as ISCs’ proliferation illustrated by Olfm4 and Ki67 (a proliferation marker) costaining on organoids (Figure 1K and Supplemental Figure 1J). The above findings implied that the pro-inflammatory cytokines in the early stages of CAC promote ISCs’ proliferation and stemness, which may contribute to tumor initiation.

### FXR agonism slows tumor progression in colitis-induced colon cancer.

**Figure 2:**
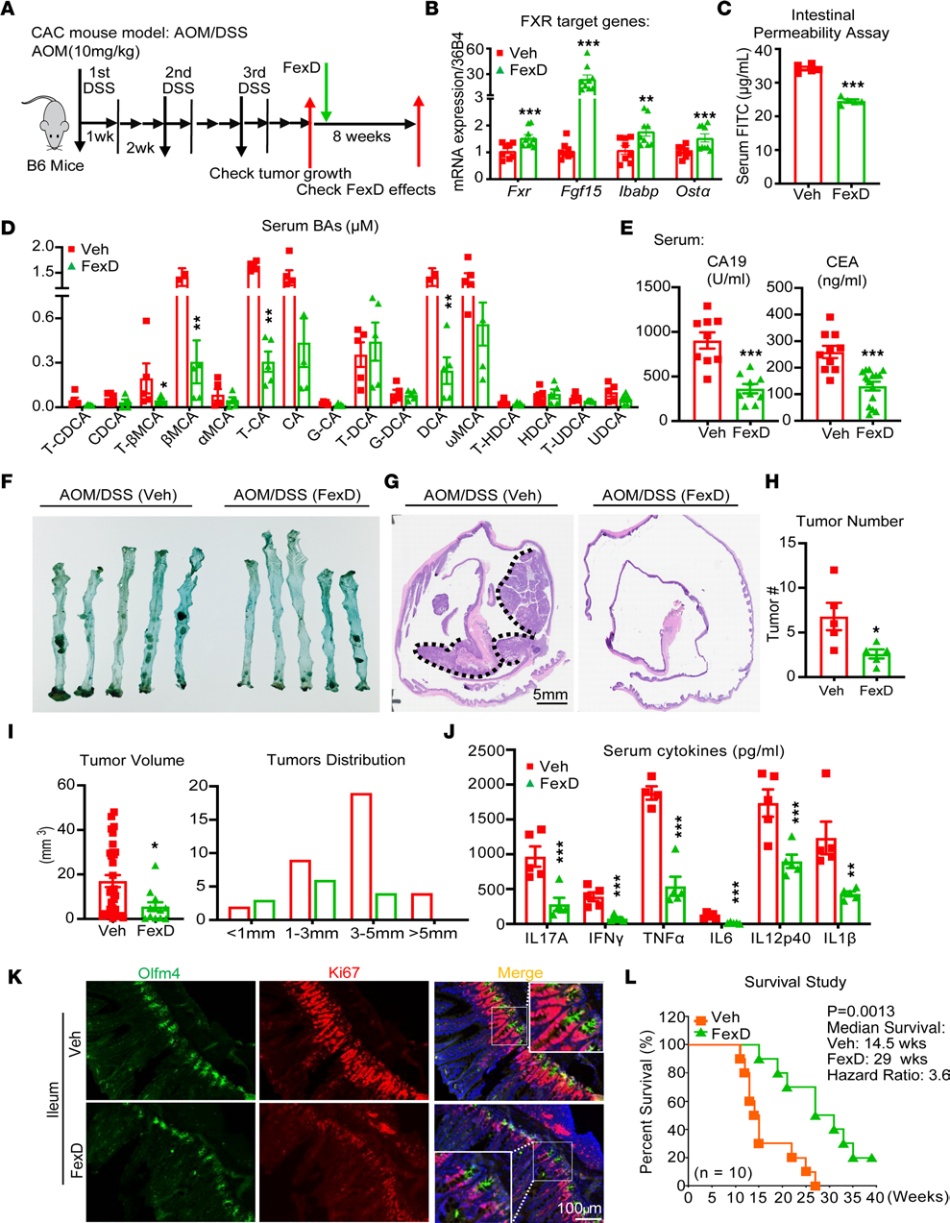
FXR agonism slows tumor progression in CAC. (**A**) The scheme of FexD treatment in CAC mice. After tumors developed, mice were treated with FexD (50 mg/kg BW/d orally) for 8 weeks, with corn oil as vehicle control. (**B**) Relative expression of FXR and its target genes (*Fgf15*, *Ibabp*, and *Ost*α) in vehicle- and FexD-treated CAC mice. (**C** and **D**) Intestinal permeability (**C**) and serum BA composition and levels (**D**) were measured in above treatment groups: glycolithocholic acid (GLCA), murideoxycholic acid (MDCA), hyodeoxycholic acid (HDCA), α-hyocholic acid (α-HCA), α-muricholic acid (α-MCA), β-MCA, ω-MCA, Tauro-β-muricholic acid (T-βMCA), and taurocholic acid (TCA). (**E**) Prognostic serum tumor markers of CRC, cancer antigen 19-9 (CA19) and carcinoembryonic antigen (CEA), in above treatment groups. (**F** and **G**) Live and H&E images of tumors in the colon. Black dot line circles the tumors. Scale bar 5 mm. (**H** and **I**) Average tumor burden (**H**), tumor volumes, and tumor size distribution (**I**) in above treatment groups. (**J**) Serum cytokine levels were measured in above treatment groups. (**K**) Co-immunostaining images of ISC marker Olfm4 (green) and proliferation gene marker Ki67 (red) in the ileum of CAC mice with FexD or vehicle treatment. Scale bar 100 μm. (**L**) Survival curves (log-rank test) for CAC mice with FexD or vehicle treatment. *n* = 3–10/group. Experiments were independently replicated twice, and representative data are shown as the mean ± SEM. *P < 0.05; **P < 0.01; ***P < 0.005, Student’s unpaired *t* test.

Since FXR signaling is compromised in both the CAC mouse model and patients with CAC, we posit that FXR activation may counteract CAC progression. To explore FXR’s function, we utilized the intestinally restricted FXR agonist, fexaramine D (FexD), in the AOM/DSS mice (21, 24, 37). Late intervention in B6 mice with FexD after the challenge of AOM/DSS profoundly restored FXR signaling (Figure 2, A and B), as evidenced by the improvement in fecal bleeding without significant changes in body weight (Supplemental Figure 2, A–C). In addition, FexD treatment prevented DSS-induced increases in intestinal permeability (Figure 2C) and reduced total BA levels and its compositional changes in both serum and fecal samples (Figure 2D and Supplemental Figure 2, C, E, and F) (38, 39). Furthermore, FexD decreased serum levels of the malignancy biomarker carcinoembryonic antigen (CEA) and the cancer antigen 19-9 (CA-19) (Figure 2E). Consistently, the histological analysis revealed that FexD profoundly reduced tumor number and size (Figure 2, F–I). In addition, FexD alleviated intestinal inflammation and changed the morphology of goblet and Paneth cells in the gut (Supplemental Figure 2, F and G). Similarly, we observed declined nuclear accumulation of β-catenin and lower expression of Ki67 in the FexD-treated group (Supplemental Figure 2H), which indicated that FexD prevented tumor progression. More importantly, FexD mitigated the systemic inflammatory responses as indicated by reduced spleen size and weight (Supplemental Figure 2I) and the levels of serum pro-inflammatory cytokines (Figure 2J and Supplemental Figure 3A). Notably, expression of *Olfm4* and *Ki67* decreased in the FexD-treated group, which indicated that FexD inhibited inflammation-induced proliferation (Figure 2K). Besides, the survival study demonstrated that FexD profoundly improved the overall survival in AOM/DSS mice. The median survival time doubled from 14.5 weeks in control group to 29 weeks in FexD group (Figure 2L). Together, FexD largely abrogated inflammation-induced tumor growth and progression in the CAC mouse model.

### FXR suppresses pro-inflammatory responses in innate and adaptive immune cells in intestinal lamina propria.

**Figure 3:**
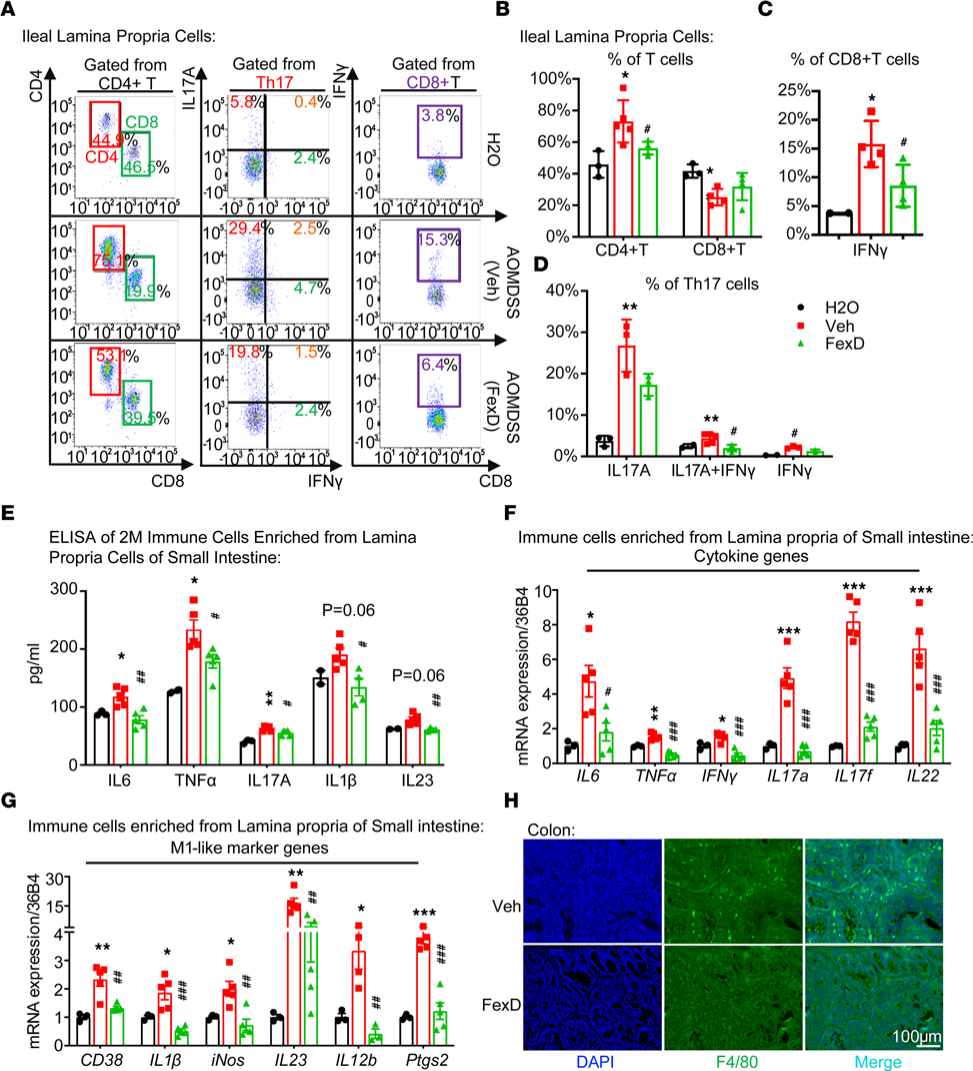
FXR suppresses pro-inflammatory response in lamina propria. (**A**) Representative flow cytometry analyses of the T cell populations in the lamina propria of ileum of CAC mice with FexD or vehicle treatment, as experimental scheme described in Figure 2A. (**B**–**D**) Quantification of CD4^+^ and CD8^+^ T cell numbers (**B**) and percentage of IFN-γ– and IL17A-secreting cells in CD8^+^ (**C**) and CD4^+^ (**D**) T cells in above treatment groups. (**E**) Indicated cytokine levels were measured in 2 million small intestinal lamina propria cells from above treatment groups. (**F** and **G**) Expression of pro-inflammatory cytokines (**F**) and M1-like marker genes (**G**) measured by quantitative reverse transcription PCR (qRT-PCR) in small intestinal lamina propria cells from above treatment groups. (**H**) Co-immunostaining images of macrophage cell marker F4/80 (green) with the nucleus counterstained with DAPI (blue) in the colon. Scale bar 100 μm. *n* = 3–5/group. Experiments were independently replicated twice, and representative data are shown as the mean ± SEM. *P* values are computed with Student’s unpaired *t* test and 1-way ANOVA test followed by multiple comparisons. *Veh versus H_2_O, ^#^FexD versus Veh; *, ^#^*P* < 0.05; **, ^##^*P* < 0.01; ***, ^###^*P* < 0.005.

Pathogenic Th17 cells, identified by secreting both IL17A and IFN-γ, have been implicated in both IBD and CAC mouse models (9, 40). The reduction of serum IL17A and IFN-γ by FXR agonism implies that FXR may impact immune cells’ function (18, 23). We analyzed potential IL17A- and IFN-γ–secreting cells enriched from intestinal lamina propria of AOM/DSS mice by FACS under later intervention of FexD treatment. Indeed, DSS induced a remarkable increase in CD4^+^ T cells, which was abrogated by FexD (Figure 3, A and B, and Supplemental Figure 3, B and C). In addition, FexD largely blocked the colitis-induced increases of IL17A^+^ and IFN-γ^+^ in CD4^+^ T cells and IFN-γ^+^ in CD8^+^ T cells in both ileal and colonic lamina propria (Figure 3, A, C, and D, and Supplemental Figure 3, B–E). Notably, FexD also decreased macrophage-secreting cytokines like IL23 and IL1β (Figure 3J). Considering that macrophages are indispensable for the induction of Th17 cells (16), we postulate that FXR modulates gut macrophages’ function and subsequently mediates Th17 response. Furthermore, FexD downregulated the pro-inflammatory cytokine production from both Th17 cells and macrophages in the ileal and colonic lamina propria cells, including IL17A and IL23 (Figure 3E and Supplemental Figure 3F), which is consistent with their mRNA level changes (Figure 3F). Furthermore, we found that FexD markedly decreased the expression of general macrophage markers, including genes associated with M1-like macrophages (Figure 3G). Besides, the macrophage numbers decreased upon FexD (Figure 3H). These data indicate that FexD not only modulates gut macrophage functions but also may inhibit macrophage recruitment to the gut.

### FXR modulates macrophage response to inflammatory insult.

**Figure 4:**
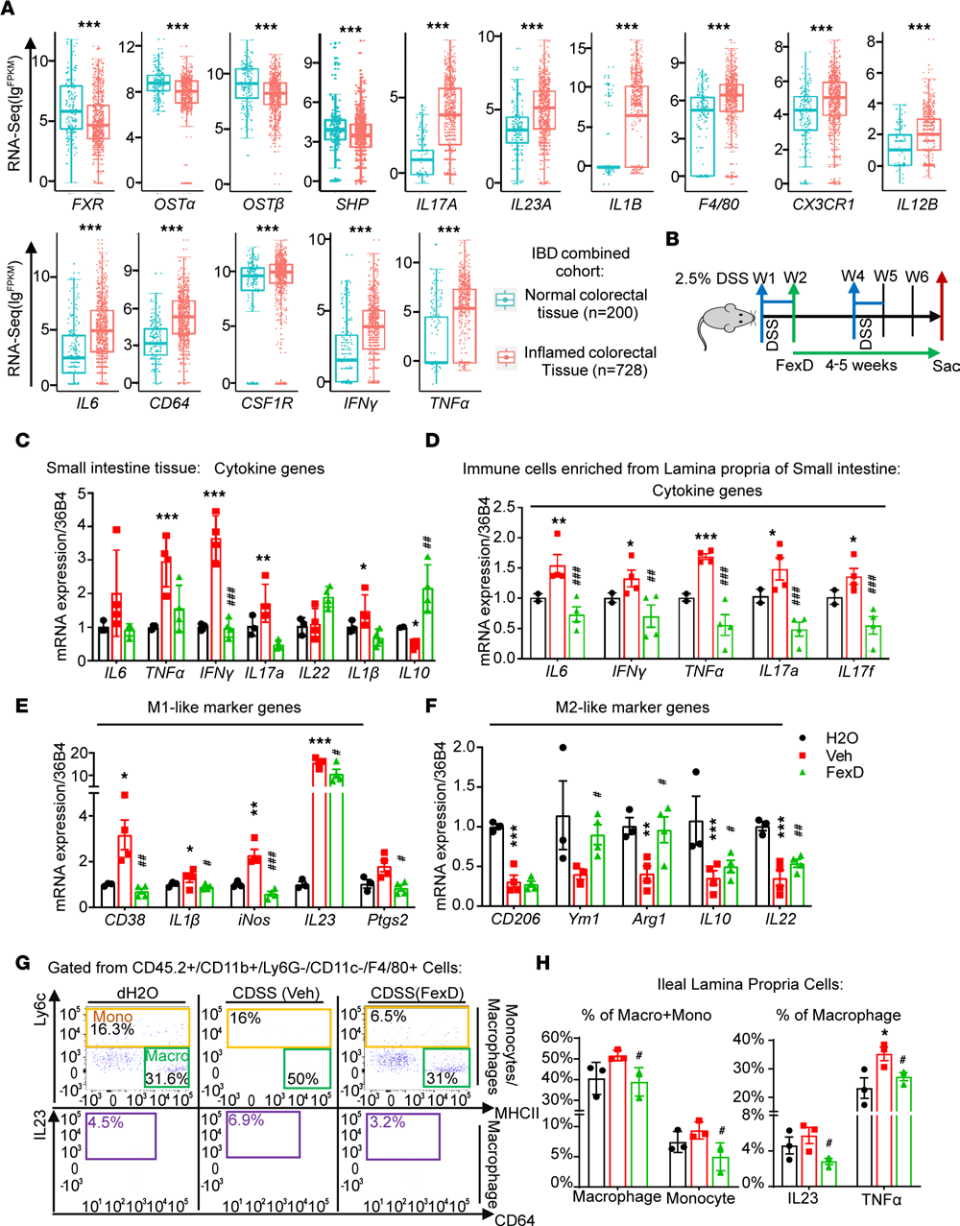
FXR modulates macrophages’ response to inflammatory insult. (**A**) Relative expression (FPKM values) of presented genes based on RNA-Seq data of normal and inflamed colorectal tissue samples from combined IBD (TaMMA) cohorts. Box plots show the interquartile range (box), median (line), and minimum and maximum (whiskers). (**B**) The scheme of FexD early intervention in WT mice under chronic DSS (CDSS) regimen or distilled water (dH_2_O) as control. After first week of CDSS administration, mice were treated with FexD (50 mg/kg BW/d orally) for 4–5 weeks, with corn oil as vehicle control. (**C**) Expression of FXR target genes in the small intestine, measured by qRT-PCR. (**D**–**F**) Expression of pro-inflammatory cytokines (**D**), M1-like macrophage cell marker genes (**E**), and M2-like macrophages marker genes (**F**) measured by qRT-PCR in small intestinal lamina propria cells from above treatment groups. (**G** and **H**) Representative flow cytometry analyses of the percentages of macrophages and monocytes and IL23^+^ and TNF-α^+^ macrophages (**G**) from small intestinal lamina propria cells. Data quantifications presented (**H**). *n* = 3–4/group. Experiments were independently replicated 3 times, and representative data are shown as the mean ± SEM. Wilcoxon test and 1-way ANOVA test followed by Tukey’s multiple comparisons are used; *CDSS versus dH_2_O, ^#^FexD versus CDSS; *, ^#^*P* < 0.05; **, ^##^*P* < 0.01; ***, ^###^*P* < 0.005.

To determine whether FXR agonism ameliorates inflammation through gut macrophages before tumor initiation, we first examined the FXR signaling, cytokine production, and macrophage marker expression in patients with IBD (transcriptome and metatranscriptome meta-analysis cohort, TaMMA cohort) (41). Consistent with CAC, expression of *FXR* and its target genes was significantly decreased, accompanied by increases in pro-inflammatory cytokines like *IL23A* and *IL1β*, as well as macrophage marker genes including *F4/80*, *CX3CR1*, and *CSF1R* (Figure 4A and Supplemental Figure 9A). The reverse correlations between *FXR* and macrophage markers indicated that FXR negatively regulated M1-like pro-inflammatory macrophages. Further, we examined the antiinflammatory role of FXR in a chronic DSS (CDSS) inflammation mouse model in which early FexD intervention started after 1 week of 2.5% DSS administration (Figure 4B). FexD alleviated morphological changes in both small intestinal and colonic epithelium, preventing immune cell infiltration compared with vehicle-treated CDSS group (Supplemental Figure 4A). This effect is attributed in part to the activation of FXR signaling, reduction in pro-inflammatory cytokines and mucous secretion in the epithelium, and restored BA homeostasis (Figure 4C and Supplemental Figure 4, B–E). We also observed a reduction of serum IL6 and IL17A levels (Figure 4D) and in their mRNA expression (Supplemental Figure 4F). Of note, we observed a decline of IL17A^+^ and IFN-γ^+^ production, specifically in Th17 cells (Supplemental Figure 4, G–I). Furthermore, FexD decreased the expression of M1-like signature genes in lamina propria cells (Figure 4E) and profoundly reduced gut macrophage and monocyte numbers, which resulted in attenuated IL23 secretion (Figure 4, G and H). Interestingly, we noticed FexD increased the expression of M2-like signature genes in lamina propria cells at this early stage (Figure 4F). Together, these findings reveal that the beneficial cascades of the effect of FXR activation in CAC may be caused by its reduction of pro-inflammatory responses in gut macrophages as early as colitis happens.

### FXR regulates BMDM polarization and functional maturation.

**Figure 5:**
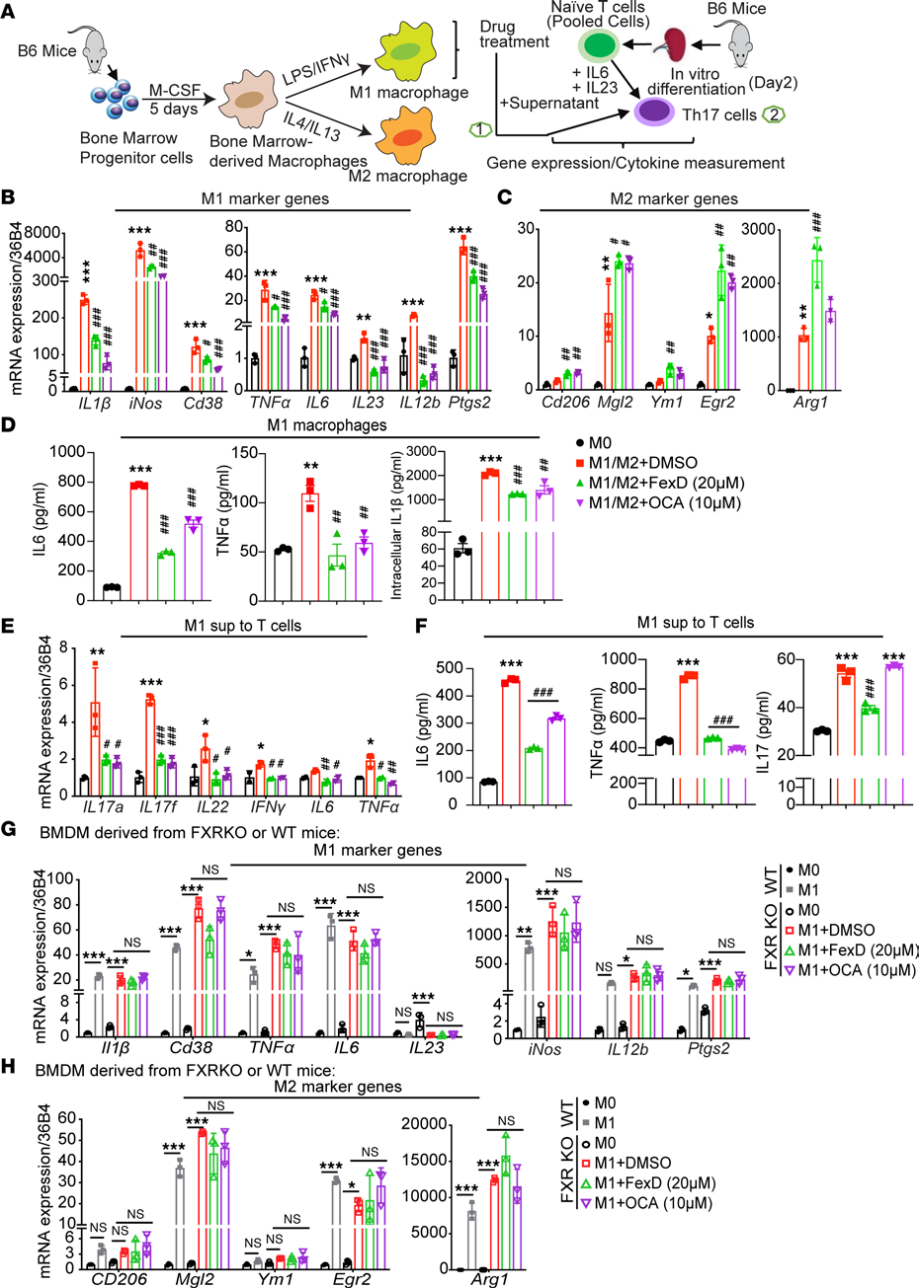
FXR regulates BMDMs’ polarization and functional maturation. (**A**) Experimental scheme of BMDM polarization and crosstalk to T cells. Polarization details are described in Methods. After polarization, BMDMs were treated with FXR agonists, FexD and OCA, or vehicle for 6 hours (step 1). Naive T cells were isolated from the spleen of WT mice and subjected to IL6 and TGF-β for initial Th17 cell in vitro differentiation for 24 hours. Then, the supernatant of BMDMs was added to Th17 cells for another 48 hours (step 2). Cell samples and cultured supernatant were harvested for qRT-PCR and ELISA. (**B** and **C**) Expression of M1 (**B**) and M2 (**C**) marker genes measured by qRT-PCR in polarized M1 or M2 BMDMs with above treatment. (**D**) Secreted (IL6, TNF-α) and intracellular (IL1β) cytokines were measured by ELISA in M1 BMDMs with above treatment. (**E**) Expression of cytokine genes measured by qRT-PCR in Th17 cells in vitro differentiated with M1 BMDM supernatant. (**F**) The level of cytokines secreted by Th17 cells was measured by ELISA. (**G** and **H**) Expression of M1 (**G**) and M2 (**H**) marker genes measured by qRT-PCR in polarized M1 or M2 FXR-deficient BMDMs. *n* = 3/group. Experiments were independently replicated 3 times, and representative data are shown as the mean ± SEM. *P* values are computed with 1-way ANOVA test followed by Tukey’s multiple comparisons. *M1/M2 versus M0 in WT or *FXR*-KO groups, ^#^FexD and OCA versus DMSO in WT or *FXR*-KO groups; *, ^#^*P* < 0.05; **, ^##^*P* < 0.01; ***, ^###^*P* < 0.005.

Circulating bone marrow–derived macrophages (BMDMs) contribute to the replenishment and maintenance of intestinal macrophages, especially during inflammation (42). To discern the impact of FXR on M1 and M2 macrophages, we induced BMDMs to differentiate into M1 and M2, followed by treatment of FXR agonists, FexD and obeticholic acid (OCA) (Figure 5A). As expected, FXR agonists dramatically decreased the expression of M1 surface markers like *CD38* and of secreted cytokines such as *IL1β* and *IL23* (Figure 5B). In contrast, FXR agonist significantly increased M2 gene expression like *CD206* (Figure 5C). These data are consistent with those in the CDSS model (Figure 4F) but not in the AOM/DSS model (Supplemental Figure 3F), which further explains that activation of FXR on M2 has different effects on colitis and CAC. In addition, FexD and OCA reduced pro-inflammatory cytokines secreted from M1 macrophages, including IL1β (Figure 5D).

Similar drug effects were observed in M1 and M2 cells polarized from RAW 264.7, a monocyte/macrophage cell line expressing FXR (Supplemental Figure 5, A–C). FXR and its target genes have limited expression in T cells (25, 27). In contrast, both BMDMs and RAW cells expressed FXR (Supplemental Figure 5D). Remarkably, FexD treatment reduced *IL17A* expression in the Th17 population (Figure 3, A–D, and Supplemental Figure 3, A–D), suggesting that the beneficial effects of FXR activation were mediated explicitly by macrophages (Figure 3, G and H).

Cytokines such as IL6, IL1β, and IL23, secreted by macrophages, could direct naive T cells to differentiate into Th17 cells in vitro (12, 14, 43). Given that FexD inhibited the function of Th17 cells in vivo, we postulate that FXR could indirectly regulate its function by modulating the crosstalk between macrophages and Th17 cells. To test this notion, we cultured the naive T cells with supernatant harvested from M1 pretreated with FXR agonists (Figure 5A). As expected, the supernatant from M1 facilitated Th17 cell differentiation, illustrated by the upregulation of Th17 cytokines like *IL17A* (Figure 5E), as well as elevated production of IL17 in Th17 cells (Figure 5F). In line with decreased M1 cytokines like IL1β and IL23 by FXR agonist administration (Figure 5D), the supernatant from M1 also inhibited Th17 cell differentiation (Figure 5, E and F). To examine whether the inhibitory effects of FXR agonistic drugs on BMDMs are FXR dependent, we generated FXR-deficient M1 and M2 macrophages by isolating BMDMs from FXR whole-body knockout mice (*FXR*-KO). As expected, FexD and OCA could not modulate M1 and M2 derived from *FXR*-KO animals (Figure 5, G and H). Taken together, the above data indicate that FXR activation enhances polarization and functional maturation of BMDMs, further leading to Th17 cells’ activation.

### Gut macrophage–intrinsic FXR senses BAs and regulates their pro-inflammatory responses.

**Figure 6:**
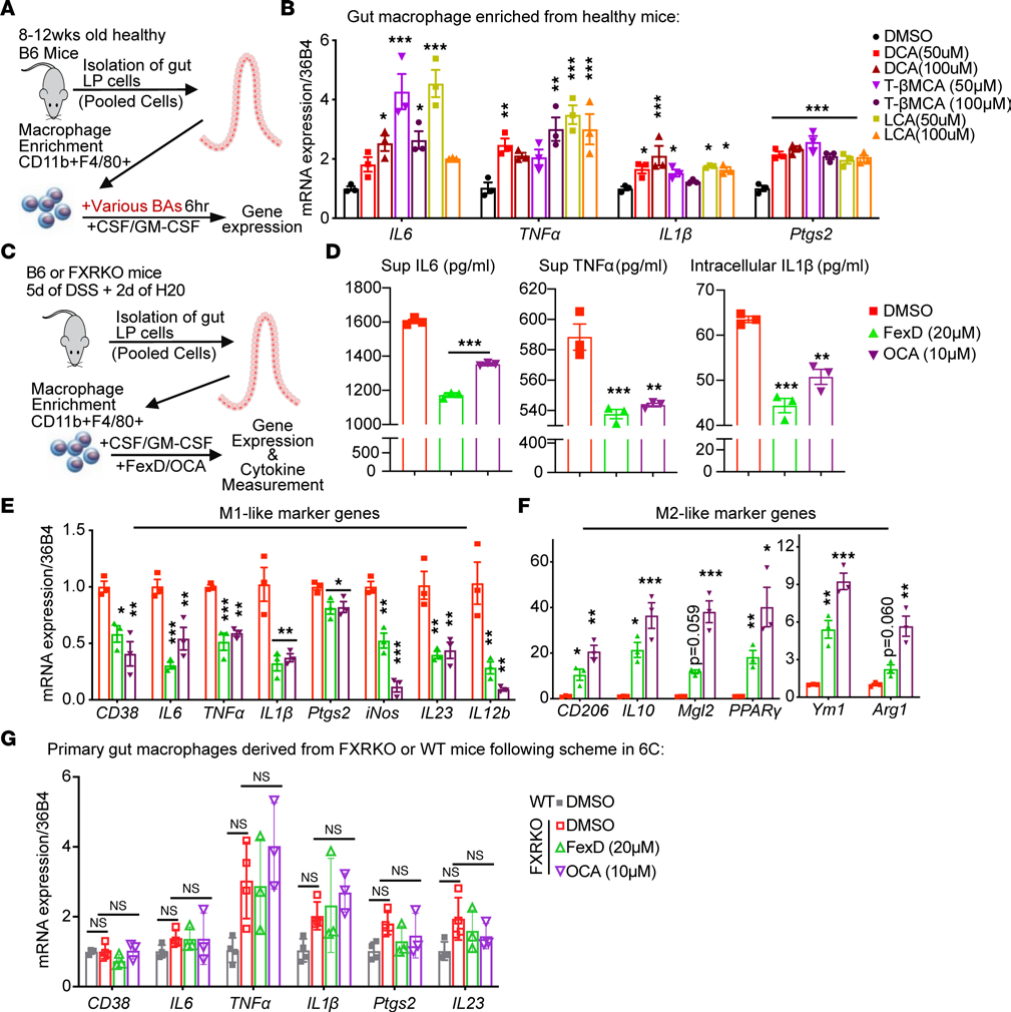
Gut macrophage–intrinsic FXR senses BA and regulates macrophage pro-inflammatory responses. (**A**) Experimental scheme of gut macrophages enriched from small intestine of WT mice and subjected to various BAs for 6 hours. (**B**) Expression of pro-inflammatory cytokines and M1-like cell marker genes measured by qRT-PCR in gut macrophages treated with a gradient concentration of indicated BAs. (**C**) Experimental scheme of gut macrophages enriched from small intestine of WT and *FXR*-KO mice under 5 days of DSS administration and 2 days of recovery, then subjected to FXR agonist treatment for 18 hours. (**D**) Secreted (IL6, TNF-α) and intracellular (IL1β) cytokines were measured by ELISA in gut macrophages. (**E** and **F**) Expression of M1-like (**E**) and M2-like (**F**) marker genes by qRT-PCR in gut macrophages with above treatment. (**G**) Expression of M1-like marker genes by qRT-PCR in FXR-deficient (*FXR*-KO) gut macrophages. *n* = 3/group. Experiments were independently replicated 3 times, and representative data are shown as the mean ± SEM. *P* values are computed with 1-way ANOVA test followed by Tukey’s multiple comparisons; *M1/M2 versus M0 in WT or *FXR*-KO groups, *P < 0.05; **P < 0.01; ***P < 0.005.

DSS-induced disruption of epithelium exposes gut macrophages to more BAs. Hence, we reasoned that gut macrophages might directly sense BAs and elicit an inflammatory response in the initial phase of CAC development (18, 44, 45). To validate this notion, we isolated and enriched the gut macrophages by a CD11b^+^F4/80^+^ bead enrichment kit from lamina propria cells of pooled healthy WT mice. Then, we maintained these gut macrophages ex vivo with CSF and GM-CSF and treated them with various BAs at the same time (Figure 6A). Interestingly, BAs that antagonize FXR activities and were aberrantly elevated in the AOM/DSS mice, such as DCA and T-βMCA (Figure 2D), could remarkably upregulate the expression of M1-like signature genes in healthy gut macrophages (Figure 6B). It suggests that downregulation of FXR signaling in gut macrophages skewed the healthy macrophages toward the M1-like status.

To further investigate the causal effect of FXR activation on the polarization of macrophages, we isolated and enriched gut macrophages from inflamed lamina propria cells of pooled DSS-administrated WT mice (Figure 6C). Then, we cultured them ex vivo with FXR agonists, FexD and OCA (Figure 6C). Indeed, FXR agonists remarkably decreased pro-inflammatory cytokine levels in gut macrophages (Figure 6D and Supplemental Figure 5E). Moreover, FXR agonists profoundly decreased the expression of M1-like signature genes such as *CD38* and *IL23*, and vastly increased the expression of M2-like marker genes like *CD206* (Figure 6, E and F), implying activation of FXR in inflamed gut macrophages skewed M1-like status toward M2-like status. In addition, flow cytometry analysis of these gut macrophages demonstrated that both FexD and OCA declined total numbers of macrophages (F4/80^+^CD11b^lo^ and CD11b^hi^ population) (Supplemental Figure 5, F–H). Furthermore, FXR agonists also significantly diminished the macrophage expression of CD64 and IL23 (Supplemental Figure 5, G and I). Interestingly, FexD and OCA also inhibited the maturation of resident macrophages (CX3CR1^+^MHCII^+^ population) ex vivo (Supplemental Figure 5, G and J). Notably, FXR agonists did not inhibit the expression of M1-like signature genes in gut macrophages isolated from DSS-administrated *FXR*-KO mice (Figure 6G), suggesting its inhibition is FXR dependent. Together, the ex vivo direct effect of FXR agonists on macrophages (Figures 5 and 6) led to the speculation that early intervention of FexD could inhibit colitis-induced pro-inflammatory macrophage and block its crosstalk to Th17 cells in vivo (Supplemental Figure 5K).

### FXR modulates macrophage-tailored intestinal immune responses to CAC.

**Figure 7:**
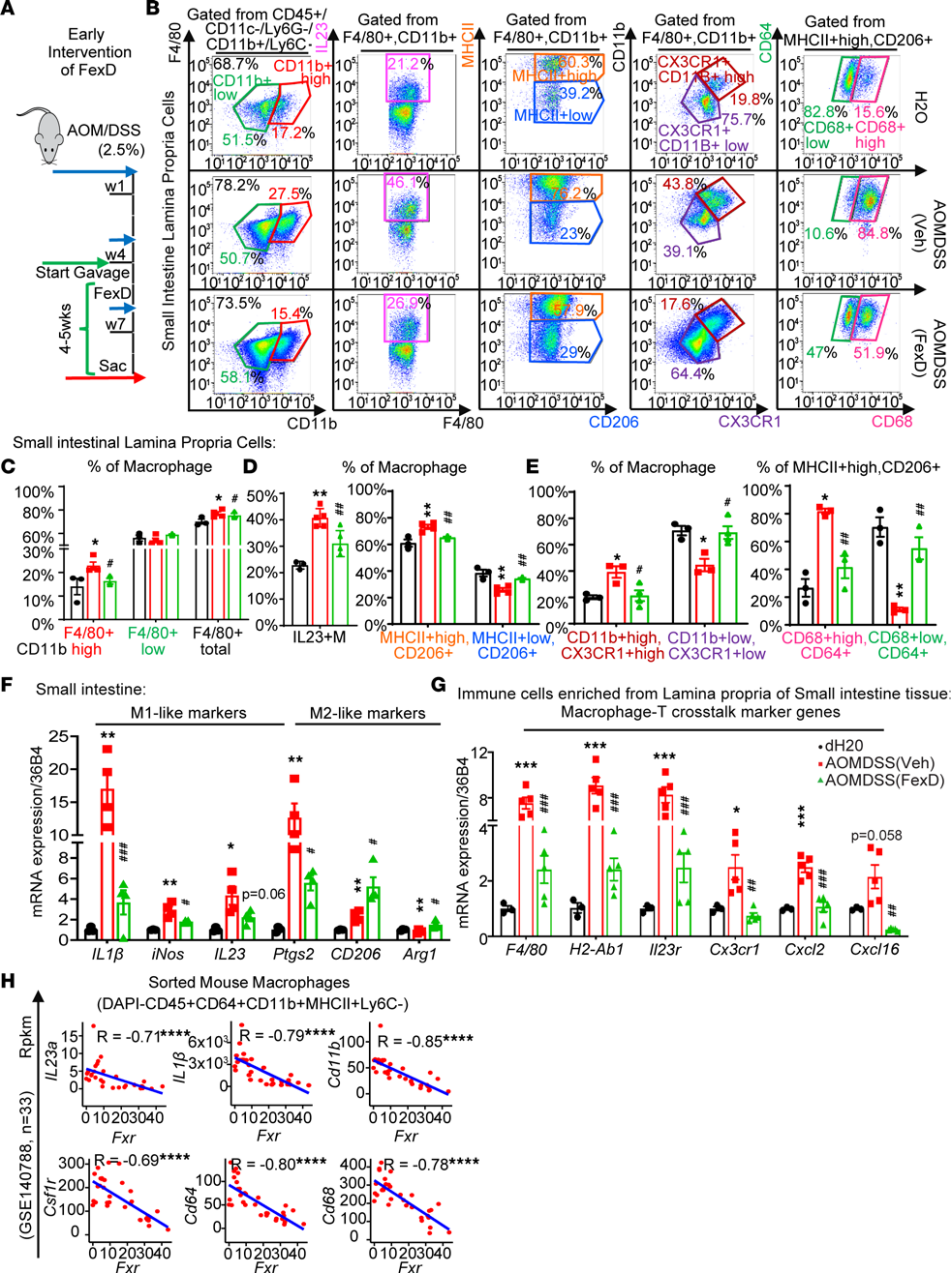
FXR mediates macrophage-tailored intestinal immune responses and homeostasis. (**A**) The scheme of FexD early intervention in WT mice under AOM/DSS regimen or dH_2_O as control. After second cycle of DSS, mice were treated with FexD (50 mg/kg BW/d orally) for 4–5 weeks, with corn oil as vehicle control. (**B**–**E**) Representative flow cytometry analyses of small intestinal lamina propria cells: CD11b^hi^F4/80^+^, CD11b^lo^F4/80^+^, and CD11b^+^F4/80^+^ total macrophages; IL23^+^ macrophages; and MHCII^hi^CD206^+^, MHCII^lo^CD206^+^, CD11b^hi^CX3CR1^hi^, CD11b^lo^CX3CR1^lo^, CD68^lo^CD64^+^, and CD68^hi^CD64^+^ macrophages (**B**). Data quantification presented (**C**–**E**). (**F**) Expression of M1-like and M2-like macrophage gene markers, measured by qRT-PCR. (**G**) Expression of macrophage surface markers like F4/80 (*Adgre1* gene), antigen-presenting proteins such as MHCII (*H2ab1* gene), IL23 receptor on CD4^+^ T cells (*IL23r* gene), and T cell recruitment chemokine genes (*Cx3cl1*, *Cxcl2*, and *Cxcl16*), measured by qRT-PCR. (**H**) Correlation between gene expression of FXR and gut macrophage markers (*IL23a*, *IL1β*, *Cd11b*, *Csf1r*, *Cd64*, *Cd68*) in an RNA-Seq database of sorted gut macrophages from B6 mice treated with 1.5% DSS with dH_2_O as a control. Rpkm, reads per kilobase million. *n* = 3–5/group. Experiments were independently replicated twice, and representative data are shown as the mean ± SEM. *P* values are computed with Student’s unpaired *t* test and 1-way ANOVA test followed by Tukey multiple comparisons. *DSS versus dH_2_O or drugs versus control, ^#^FexD versus vehicle in AOM/DSS cohort; *, ^#^*P* < 0.05; **, ^##^*P* < 0.01; ***, ^###^*P* < 0.005; ****P < 0.001.

To test this possibility, AOM/DSS mice were treated with early intervention of FexD after the first cycle of DSS, and the gut macrophages from lamina propria cells were analyzed (Figure 7A). Indeed, colitis significantly increased the total number of intestinal and colonic macrophages (F4/80^+^CD11b^lo^ and CD11b^hi^ populations), which were reduced by early intervention of FexD (Figure 7, B and C, Supplemental Figure 6A, and Supplemental Figure 7C), indicating that FexD inhibited macrophage recruitment and maturation. Furthermore, FexD profoundly reduced the expression of IL23 and the percentage of MHCII^+^CD206^+^ macrophages in AOM/DSS mice (Figure 7, B and D). Nonetheless, we observed an increase in CD11b^hi^CX3CR1^hi^ population in vehicle-treated AOM/DSS mice, but the increase was eliminated with FexD (Figure 7, B and E), which demonstrated that inflammation stimulated monocytes’ recruitment and gut macrophages’ maturation. Of note, even in the M1-like MHCII^+^CD206^+^ cells, we noticed a CD64^+^CD68^hi^ population rise in vehicle-treated AOM/DSS mice, but this was declined by FexD (Figure 7, B and E), which might be the most pro-inflammatory M1-like cells. All above data indicate that FexD treatment skewed M1-like status toward M2-like status in the inflamed gut. This observation was substantiated by the expression of signature genes involved in crosstalk between macrophages and T cells. FexD strikingly decreased the gene expression of macrophage surface markers like F4/80, antigen-presenting proteins like MHCII and IL23 receptor on CD4^+^ T cells, as well as T cell recruitment chemokine genes (Figure 7G) (8, 46). Notably, we detected similar effects of FexD in colonic and splenic immune cells of AOM/DSS mice compared to the control group (Supplemental Figure 6, G–J). However, the percentage of F4/80^+^CD11b^hi^ macrophages and CD11b^hi^CX3CR1^hi^ macrophages in the spleen (Supplemental Figure 6, G–J) was significantly smaller compared with in the ileum and colon (Figure 7, A–E, and Supplemental Figure 6, A–F). Taken together, these data indicate that FXR modulates the recruitment and maturation of gut macrophages, including both monocyte-derived and tissue-resident macrophages (42, 47).

To further validate FXR as a key regulator of gut macrophages, we examined the correlation between FXR signaling and macrophage markers in an RNA-Seq database of sorted gut macrophages from WT mice treated with 1.5% DSS (48). As expected, expression of *Fxr* was inversely correlated with that of macrophage pro-inflammatory cytokines such as *IL23a* and *IL1β* (Supplemental Figure 6J) and surface marker genes like *Csf1r* and *Cd64* (Figure 7G). More important, parsing patients from The Cancer Genome Atlas (TCGA) CRC database, patients expressing higher levels of macrophage pro-inflammatory signature genes, which are negatively regulated by FXR, had worse outcome as compared with lower expressors (Supplemental Figure 9B). This underscores the contribution of gut macrophages to CRC and suggests that FXR may elicit cancer-preventive effects by modulating gut macrophages.

### FXR attenuates gut macrophages’ responses to inflammatory insults.

**Figure 8:**
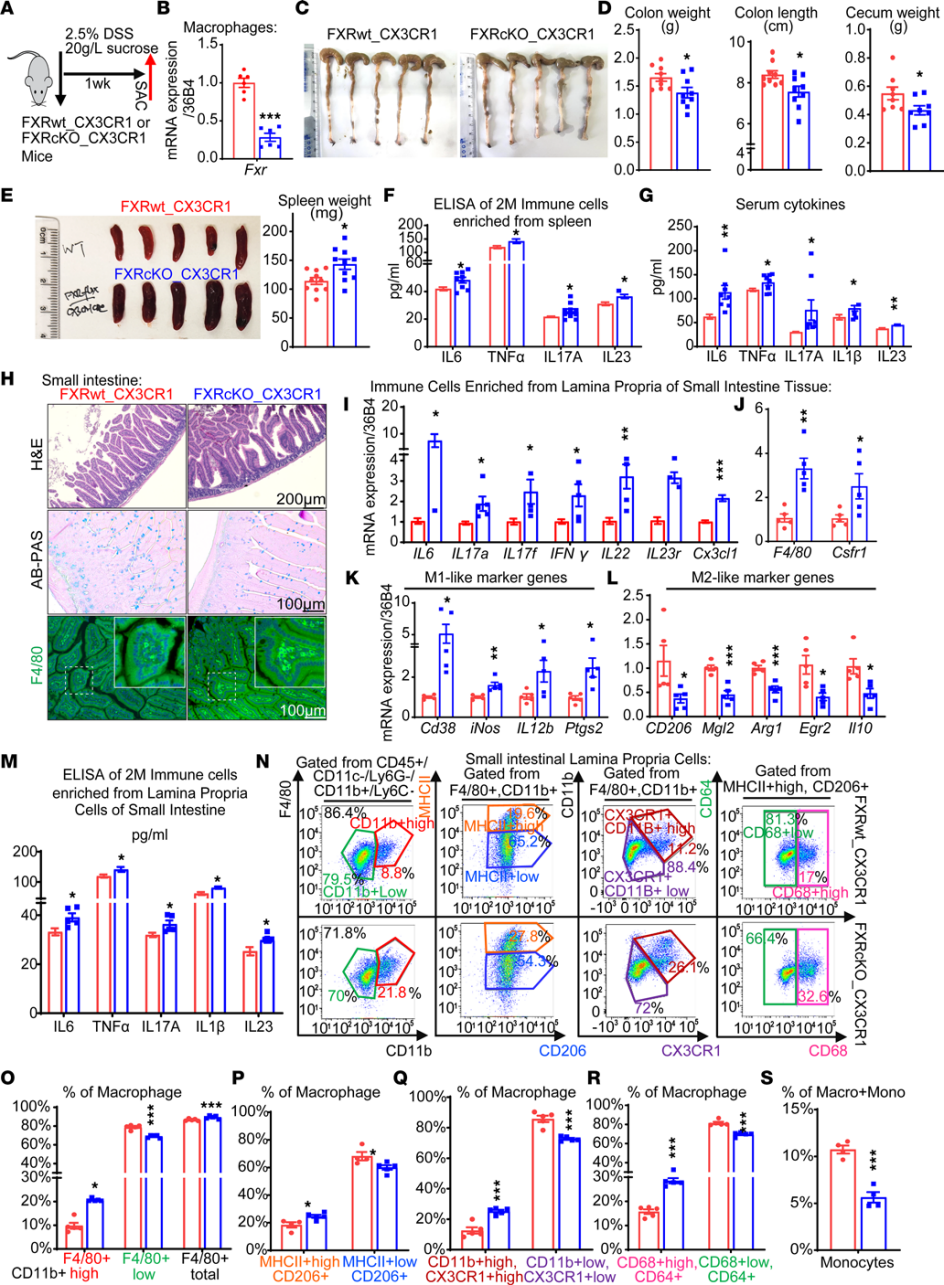
FXR attenuates gut macrophages’ responses to inflammatory insults. (**A** and **B**) Experimental scheme of WT (*FXR*wt_*CX3CR1* group) and FXR conditional knockout in CX3CR1^+^ cells (*FXR*cKO_*CX3CR1* group) mice challenged by CDSS (**A**). The deletion of FXR in CX3CR1^+^ cells was checked by FXR expression in enriched gut macrophages (**B**). (**C** and **D**) Representative live images of colon and quantification of colon weight and length and cecum weight in *FXR*wt_*CX3CR1* group and *FXR*cKO_*CX3CR1* group. (**E** and **F**) Representative live images of spleen and quantification of spleen weight (**E**) and pro-inflammatory cytokine levels of 2 million isolated splenic immune cells (**F**) in *FXR*wt_*CX3CR1* and *FXR*cKO_*CX3CR1* group. (**G**) Serum cytokines in *FXR*wt_*CX3CR1* group and *FXR*cKO_*CX3CR1* group. (**H**) Representative H&E staining, Alcian blue (AB)/periodic acid–Schiff (PAS) staining, and F4/80 staining of macrophages of small intestine in *FXR*wt_*CX3CR1* and *FXR*cKO_*CX3CR1* group. (Scale bar 100 or 200 μm.) (**I**–**L**) Expression of general pro-inflammatory cytokine genes (*IL6*, *TNFα*, *IL17A*, etc.) and T cell marker genes (*IL23r*, *Cx3cl1*) (**I**), general macrophage marker genes (*F4/80*, *Csfr1*) (**J**), M1-like macrophage signature genes (*Cd38*, *iNos*, etc.) (**K**), and M2-like macrophage signature genes (*Mgl2*, *Arg1*, etc.) (**L**) in immune cells isolated from the lamina propria of the small intestine, measured by qRT-PCR. (**M**) Levels of pro-inflammatory cytokines in immune cells isolated from the lamina propria of the small intestine in *FXR*wt_*CX3CR1* and *FXR*cKO_*CX3CR1* group. (**N**) Representative flow cytometry analyses of small intestinal lamina propria cells: CD11b^hi^F4/80^+^, CD11b^lo^F4/80^+^, and CD11b^+^F4/80^+^ total macrophages and MHCII^hi^CD206^+^, MHCII^lo^CD206^+^, CD11b^hi^CX3CR1^hi^, CD11b^lo^CX3CR1^lo^, CD68^lo^CD64^+^, and CD68^hi^CD64^+^ macrophages. (**O**–**S**) Data quantification of macrophages, subtypes of macrophages, and monocytes corresponding to above flow analysis. Experiments were independently replicated 2 times, and representative data are shown as the mean ± SEM. *FXRwt_*CX3CR1* versus *FXR*cKO_*CX3CR1*. *P < 0.05; **P < 0.01; ***P < 0.005, Student’s *t* test (unpaired).

To further investigate the protective role of FXR, we transplanted WT mice with *FXR*-KO bone marrow (*FXR*-KOBM) and used *FXR*wt bone marrow as controls (Supplemental Figure 7A). An exaggerated acute DSS phenotype was evident in *FXR*-KOBM mice, including increased total serum BA levels, elevated gene expression of pro-inflammatory cytokines and M1-like macrophage markers, and decreased M2-like macrophage markers in intestinal immune cells (Supplemental Figure 7, B–F). Furthermore, knockout of FXR profoundly increased the macrophage numbers, and the percentage of MHCII^+^CD206^+^ cells, as well as macrophages’ production of IL23 and TNF-α (Supplemental Figure 7, G and H). Furthermore, in a specific *FXR*-flox/*CX3CR1*-cre model where FXR was conditionally knocked out (*FXR*-cKO) in gut macrophages (Figure 8, A and B), FXR deletion dampened the DSS-induced gut phenotypes, such as shortened colon length and decreased cecum weight (Figure 8, C and D). In addition, FXR conditional deletion resulted in enlarged spleen size and weight (Figure 8E) and elevated pro-inflammatory cytokines in both splenic cells and serum samples (Figure 8, F and G). Of note, intestinal tissue in *FXR*-cKO group also displayed more immune cells’ infiltration, irregular-shaped Paneth and goblet cells, and accumulated gut macrophages, compared with *FXR*wt group (Figure 8H). Furthermore, expression of pro-inflammatory cytokines (*IL6*, *IL17A*, etc.), general macrophage surface markers (*F4/80*, *Csfr1*), and M1-like macrophage signature genes (*Cd38*, *iNos*, etc.) was significantly enhanced, alongside diminished expression of M2-like macrophage marker genes (*Mgl2*, *Arg1*, etc.) (Figure 8, I–L). Similar increases of above pro-inflammatory cytokines were also observed in *FXR*-cKO group, including IL23 (Figure 8M). Flow cytometry analysis of lamina propria cells isolated from both small intestinal and colon sections also displayed an increment of total macrophages (F4/80^+^CD11b^+^) and subpopulations of gut macrophages (MHCII^hi^CD206^+^, CD11b^hi^CX3CR1^hi^, CD68^+^CD64^+^) and reduction of monocytes (Figure 8, N–S, and Supplemental Figure 8). These data together implied the intrinsic modulation role of FXR in gut macrophage maturation and activation.

In summary, the above findings support a crucial role of FXR in the differentiation and functional maturation of gut macrophages in response to inflammatory stimuli and the subsequent crosstalk to Th17 cells, unveiling the potential of FXR as a therapeutic target in intestinal inflammatory diseases such as CAC.

## Discussion

Carcinogenesis is triggered by cell-intrinsic and -extrinsic risk factors that promote cell proliferation, resistance to apoptosis, genomic instability, and reprogramming of stromal and immune environments (4, 36). Chronic inflammation is a prolonged disruption of tissue homeostasis, which promotes all stages of tumorigenesis (2, 4, 49). Therefore, CAC engages well-orchestrated interactions of host epithelial, stromal, and immune cells, as well as luminal gut microbes and their products, which is a fantastic model to study inflammatory tumor microenvironment (50). Our results provide compelling evidence of FXR’s role in regulating inflammatory responses through gut macrophages and its mediated Th17 responses in CAC.

As early dietary sensors and genetic effectors, BAs have emerged as pleiotropic signaling molecules important in maintaining gut homeostasis through regulation of barrier integrity and repair, antimicrobial defense, and mucosal immunity (5, 18, 24, 46). Notably, BA levels are increased in CAC, in concert with decreased FXR signaling. Reciprocally, FXR agonists reinstate BAs’ homeostasis, restore epithelial permeability, and reduce intestinal inflammation (21, 24, 39). Moreover, the protective effects of FXR activation account for the attenuation of macrophages and Th17 pro-inflammatory responses in CAC.

Gut-resident macrophages can be derived and differentiated from monocytes in response to local environmental cues. Gut macrophages respond to extracellular bacteria and their products while maintaining tolerance to intestinal commensals (26, 27, 51–54). Interestingly, we discovered that gut macrophages also sense BAs, derived from both the host and the microbes (Figure 5). Additionally, we demonstrate that FXR agonists reduce inflammatory cytokines by directly affecting the differentiation and functional maturation of macrophages (Figure 5). Of note, monocyte- and macrophage-derived cytokines, such as IL23 and IL1β, function as early effectors, thereby initiating intestinal inflammation (44).

Sustained activation of innate immune responses, due to the lasting impaired BA homeostasis, may drive progression toward chronic colitis. Key orchestrators include recruited monocytes and macrophages, which produce a range of pro-inflammatory cytokines that shape pathologic T cell responses (5, 30–32, 43, 55–57). In both humans and mice, activation of tissue-resident macrophages and sustained production of IL23, IL1β, and IL12 can switch barrier-promoting Th17 responses into a pathogenic mode, leading to elevated IL17A, IFN-γ, and IL17F (8, 10–12, 43, 55, 56, 58). Consistently, our results show that FexD reshaped pathogenic Th17 cells’ function in vivo, and FXR agonists blocked the Th17 cells’ maturation through macrophage-mediated crosstalk (Figures 3, 5, and 7, and Supplemental Figures 3 and 5). Indeed, FexD inhibits the activation and recruitment of gut macrophages in vivo, evidenced by the reduced number of total resident macrophages, and lower expression of M1-like cells, as well as recruitment of fewer monocyte-derived macrophages in the gut (Figure 7 and Supplemental Figure 6). Intriguingly, FexD also impedes effector T cells’ activation by blocking the abilities of antigen presentation and chemokine secretion in gut macrophages (Figure 7). Although macrophages, due to their exceptional plasticity during colitis and cancer progression, could be pleiotropic orchestrators for pro- or antitumor immunity (17, 29), IL23 and IL17A are known promoters of early CRC (9, 12). Thus, our study mainly focuses on the FXR agonism mechanism of actions by regulating gut macrophages at the early pro-inflammatory stage of CAC progression.

Intestinal homeostasis is sustained by interactions between epithelial and immune cells subjected to cytokine regulation. Pro-inflammatory cytokines enhanced at the early stage of CAC can lead to excessive tissue regeneration, triggering the proliferation and clonal expansion of initiated tumor cells (44, 50). Moreover, cytokines’ functions are context dependent and can exert opposing effects depending on the degree of inflammation (59). Thus, we delineated the impact of CAC-enriched cytokines on ISCs’ proliferation using primary intestinal organoids (Figure 1), with the goal of mimicking inflammation-predisposed tumorigenesis. Indeed, stimulation of intestinal organoids with pivotal cytokines from M1 macrophages and pathogenic Th17 cells facilitates ISCs’ renewal and differentiation (Figure 1 and Supplemental Figure 1).

Cytokine-targeted therapies, including anti–TNF-α, anti-IL17A, and anti-IL23, represent major advances in reducing intestinal inflammation, despite relapse and failure to respond. Recently, the clinical effects of FXR agonists such as OCA have been explored extensively in liver steatosis and cirrhosis (22, 23). Our studies suggest that pharmacologic FXR activation may prevent IBD and CAC progression through its synergistic effects in IECs and immune cells.

## Methods

### Animal experiments.

C57BL/6J (WT; catalog 000664) mice were purchased from Jackson Laboratory. FXR whole-body KO mice (*FXR*-KO) and FXR-specific deletion mice (*FXR*^fl/fl^) were gifted from Ronald Evans’s lab (Salk Institute). *CX3CR1*-Cre mice (catalog 025524) were purchased from Jackson Laboratory. All the mice were maintained on a normal chow diet (LabDiet 5001). To induce classic CAC in WT mice, a single intraperitoneal injection of mutagen AOM (10 mg/kg body weight) and 3 cycles of inflammatory agent DSS (2.5%) each for 5–7 days in drinking water were given to the WT mice within approximately 9 weeks in total. We added 20 g/L sucrose into the DSS water to reduce its bitterness, in order to keep the similar water intake of each mouse per day, per detailed scheme in Supplemental Figure 1A. For drug treatment, FexD (50 mg/kg in corn oil) or vehicle was started orally gavaged daily on mice from 8 weeks of age. For late intervention, FexD or vehicle treatment were started at the end of 9 weeks of AOM/DSS administration and continued for 8–12 weeks; see experimental scheme in Figure 2A. For early intervention in AOM/DSS mice, FexD or vehicle was started at second cycle of DSS administration and continued at same time with the third cycle of DSS for 4–5 weeks; see experimental scheme in Figure 7A. We utilized these 2 schemes of FexD treatment, considering tumor-associated macrophages may already form in the late intervention, whereas pro-inflammatory macrophages may only occur at early intervention time point. To study inflammation only, WT mice under CDSS regimen (2.5% DSS in drinking water) were utilized. One week after, early intervention of FexD was started and continued for 4–5 weeks before mice were sacrificed; see Figure 4B.

### Fecal occult blood test, intestinal permeability, and histology examination.

The fecal occult blood test was used to check for the presence of blood in the stool (Beckman Coulter, catalog 60151A). Intestinal permeability was measured by oral FITC-dextran leakage into blood in mice. Briefly, mice were orally gavaged with 150 μL of 100 mg/mL 4 kDa FITC-labeled dextran (FD4, MilliporeSigma) in PBS 4 hours before sacrifice. FITC-derived fluorescence was quantified in the serum using a Varioskan LUX Multimode Microplate Reader (Thermo Fisher Scientific). Concentrations were determined using a standard curve generated by serial dilution of FITC-dextran. Swiss-rolled sections of mouse intestine subjected to H&E staining were used for tumor stage examination (Pacific Pathology, UCSD Histopathology Services, and UWCCC Histology Lab). Images were taken using Olympus Virtual Slide Microscope VS120.

### Total BA measurement and BA composition analysis.

Total BAs in mouse serum and fecal samples were measured by the Total Bile Acid Assay Kit (Diazyme Laboratories, catalog DZ042A-K) according to the manufacturer’s instructions. Serum samples were diluted at 1:5 with a blank buffer, and calculations were performed using standard controls included in the kit. For fecal samples, total fecal BAs were extracted from 2 g feces pooled from 10 mice from 2 cages (5 mice per cage) under one arm of treatment. Authentic BA standards were purchased from MilliporeSigma, except GLCA, MDCA, HDCA, α-HCA, α-MCA, β-MCA, ω-MCA, and T-βMCA, which were purchased from Steraloids. TCA was purchased from Calbiochem, and the deuterated BA standards cholic-2,2,4,4-d4 acid, chenodeoxycholic-2,2,4,4-d4 acid, and lithocholic-2,2,4,4-d4 acid were purchased from C/D/N Isotopes. Mouse serum (20 μL) was protein precipitated with 80 μL of ice-cold acetonitrile containing 3.28 ng of deuterated cholic acid (2, 2, 4, 4-d4 cholic acid) as an internal standard, vortexed 1 minute, and centrifuged at 9,500*g* for 10 minutes at 4°C. The supernatant was evaporated under vacuum at room temperature, reconstituted in assay mobile phase, and transferred to a 96-well plate for analysis. A Nexera UPLC (SHIMADZU) system was used in combination with a QTRAP 5500 mass spectrometer (SCIEX) with Analyst Software 1.6.2 (60). Chromatographic separations were performed with an ACQUITY (Waters) UPLC BEH C18 column (1.7 μm, 2.1 × 100 mm). The temperatures of the column and autosampler were 65°C and 12°C, respectively. The sample injection was 1 μL. The mobile phase consisted of 10% acetonitrile and 10% methanol in water containing 0.1% formic acid (mobile phase A) and 10% methanol in acetonitrile 0.1% formic acid (mobile phase B) delivered as a gradient: 0–5 minutes mobile phase B held at 22%; 5–12 minutes mobile phase B increased linearly to 60%, 12–15 minutes mobile phase B increased linearly to 80%, and 15–19 minutes mobile phase B constant at 80% at a flow rate of 0.5 mL/min. The mass spectrometer was operated in negative electrospray mode working in the multiple reaction mode (MRM). Operating parameters were curtain gas 30 psi, ion spray voltage 4,500 V, temperature 550°C, ion source gas 160 psi, and ion source gas2 65 psi. Transition MRMs, declustering potential, entrance potentials, and collision cell exit potentials were optimized using the Analyst software. Dwell times were 25 ms.

### Cytokine and cancer tumor marker measurement.

Serum levels of mouse cytokines were analyzed by the Luminex Bio-Plex system. Cell culture supernatant cytokines were measured by the Varioskan LUX Multimode Microplate Reader (Thermo Fisher Scientific). The mouse cytokine 23-multiplex assay was carried out according to the manufacturer’s instructions (Bio-Rad, catalog M60009RDPD). Specific cytokines such as IL23, IL6, IL1β, TGF-β, TNF-α, and IL17A were measured with corresponding cytokine ELISA kits (Thermo Fisher Scientific, catalog 88-7230-88, catalog 88-7064-88, catalog 88-7013-88, catalog 88-8350-88, catalog 88-7324-88, and catalog 88-7371-88). In addition, tumor markers CEA (Lifespan Biosciences Inc, catalog LS-F5042) and CA 19-9 (Lifespan Biosciences Inc, catalog LS-F24309) were used to distinguish between benign and malignant tumors.

### Isolation and generation of intestinal organoid.

Intestines were washed in ice-cold PBS (Mg2+/Ca2+) (Corning, catalog 21-031-CM) containing 2% BSA (Gemini Bio-products, catalog 900-208) and 2% antibiotic-antimycotic (Gibco, catalog 15240-062). Crypts and villi were exposed by dicing the intestines into small pieces (1–2 cm long), followed by extensive washes to remove contaminants (61). Then, a gentle cell dissociation reagent (Stemcell Technologies, catalog 7174) was used according to the manufacturer’s instructions. Briefly, intestinal pieces were incubated on a gently rotating platform for 15 minutes. After that, the gentle cell dissociation reagent was removed, and the intestines were washed 3 times with PBS wash buffer with vigorous pipetting. The first and second fractions that usually contain loose pieces of mesenchyme and villi were not used. Fractions 3 and 4 containing the intestinal crypts were collected and pooled. Isolated crypts were filtered through a 70 μm nylon cell strainer (Falcon, Corning catalog 352350). Crypts were counted, then embedded in Matrigel (Corning, growth factor reduced, catalog 354230) and cultured in the Intesticult organoid growth medium (Stemcell Technologies, catalog 6005).

### Cell lines and organoid studies.

The mouse macrophage cell line RAW 264.7 (ATCC TIB-71) was acquired and cultured according to the supplier’s instructions. FexD, OCA, and GW4064 were dissolved in DMSO. FexD was used as a custom production (Wuxi Biologics), and OCA and GW4064 were purchased from Cayman Chemicals. Organoids were treated with drugs on day 2 or day 3 after plating to capture the early growth phase. Images of organoid morphology changes after drug treatment were taken with EVOS M5000 microscope (Thermo Fisher Scientific). CellTiter-Glo Luminescent 3D Cell Viability Assay Kit (Promega, catalog G9683) was used to check the cell viability after drug treatment. Organoids were directly lysed using TRIzol reagent (Ambion, catalog 15596026), and RNA was extracted with RNeasy Mini Kit (QIAGEN, catalog 74106).

### Immunofluorescence staining of mouse intestinal organoids.

Intestinal organoids growing in 8-well Chamber slides (Thermo Fisher Scientific, catalog 154534) were fixed with 300 μL 4% paraformaldehyde at room temperature for 20 minutes. After 20 minutes, organoids were washed once with immunofluorescence (IF) buffer (PBS containing 0.2% Triton X-100 and 0.05% Tween) and permeabilized with 300 μL permeabilization solution (PBS containing 0.5% Triton X-100) at room temperature for 20 minutes. After permeabilization, organoids were washed once with IF buffer and blocked with blocking solution (IF buffer with 1% BSA) at room temperature for 30 minutes. Organoids were then incubated with primary antibodies (anti-Ki67, 1:100, Cell Signaling Technology [CST], catalog 9449s; anti-Olfm4, 1:100, CST, catalog 39141s) diluted in 200 μL blocking solution for overnight at 4°C. Organoids were washed 3 times with 300 μL IF buffer (5 minutes each wash) on the next day and incubated with secondary antibodies (Alexa Fluor 488–conjugated anti-mouse IgG, 1:200, CST 4408s; Alexa Fluor 555–conjugated anti-rabbit IgG, 1:200, CST 4413s) prepared with 200 μL blocking solution at room temperature in the dark for 60 minutes. After the secondary antibody incubation, organoids were washed 3 times with IF buffer and covered with 20 μL antifade mounting medium with DAPI (Vector Laboratories, catalog H-1200-10), and the sections were sealed with nail polish.

The 10× and 20× images were taken with EVOS M5000 fluorescence microscope, and 40× and 60× images were taken with Olympus FV3000 laser scanning confocal microscope with FV31S-SW software.

### IF staining of paraffin-embedded mouse tissue.

Tissue sections were deparaffinized and rehydrated via a sequential incubation with Histo-Clear (Thermo Fisher Scientific) (3 times for 5 minutes), 100% ethanol (5 minutes), 75% ethanol (5 minutes), 50% ethanol (5 minutes), 25% ethanol (5 minutes), and tap water (2 times for 5 minutes). We submerged the sections in sodium citrate buffer (10 mM, pH = 6, 0.05% Tween) and placed them in a pressure cooker, setting the pressure high for 10 minutes for antigen retrieval. We let the slides cool at room temperature. Next, we incubated the sections at room temperature for 20 minutes with permeabilization buffer (PBS containing 0.5% Triton X-100). Sections were then blocked with blocking solution (IF buffer with 5% BSA) at room temperature for 60 minutes. After blocking, we incubated the sections with primary antibodies (anti-Ki67, 1:100, CST, catalog 9449s; anti-Olfm4, 1:100, CST, catalog 39141s; anti–β-catenin, 1:100, CST, catalog 8480s; anti-F4/80, 1:50, BioLegend, catalog 123102) overnight in IF buffer containing 1% BSA. The sections were washed 3 times with IF buffer containing 1% BSA and incubated with secondary antibodies (Alexa Fluor 488–conjugated anti-mouse IgG, 1:200, CST 4408s; Alexa Fluor 555–conjugated anti-rabbit IgG, 1:200, CST 4413s) prepared in IF buffer containing 1% BSA at room temperature in the dark for 60 minutes. After secondary antibody incubation, we washed each section 3 times with 200 μL IF buffer. We covered the section with 20 μL antifade mounting medium with DAPI and sealed the sections with nail polish. Images were taken with EVOS M5000 fluorescence microscope.

### PAS/Alcian blue staining.

Tissue sections were deparaffinized and rehydrated via a sequential incubation with Histo-Clear (3 times for 5 minutes), 100% ethanol (5 minutes), 75% ethanol (5 minutes), 50% ethanol (5 minutes), 25% ethanol (5 minutes), and tap water (2 times for 5 minutes). We submerged sections in Alcian blue (pH 2.5, 1% in 3% acetic acid, EMS, catalog 26026-13) at room temperature for 30 minutes. The sections were rinsed in tap water 3 times and incubated with 0.5% periodic acid (EMS, catalog 19324-05) at room temperature for 15 minutes. We rinsed the sections in tap water 3 times and incubated them with Schiff’s reagent (VWR, I470302-348) at room temperature for 45 minutes. After 45 minutes, we rinsed the sections with tap water and dipped the sections into hematoxylin for 5 seconds, then washed in tap water again. We dipped the sections 5 times into 0.01N hydrochloric acid, then rinsed with tap water. We dehydrated the sections through a serial incubation with 90% ethanol (2 times for 5 minutes), 100% ethanol (2 times for 5 minutes), and Histo-Clear (3 times for 5 minutes). We dried the sections, covered them with glycerol, and sealed them with nail polish. Images were taken with EVOS M5000 microscope.

### H&E staining.

Tissue sections were deparaffinized and rehydrated via a sequential incubation with Histo-Clear (3 times for 5 minutes), 100% ethanol (5 minutes), 75% ethanol (5 minutes), 50% ethanol (5 minutes), 25% ethanol (5 minutes), and tap water (2 times for 5 minutes). We submerged the sections into the hematoxylin solution (Ricca Chemical Company, catalog 353732) for 45 seconds, then rinsed with deionized water (2 minutes) and tap water (2 times for 5 minutes). We blotted excessive water from the sections and dipped into the eosin solution for 45 seconds (Ricca Chemical Company, catalog 284532). We dehydrated the sections through a serial incubation with 90% ethanol (2 times for 2 minutes), 100% ethanol (2 times for 2 minutes), and Histo-Clear (3 times for 2 minutes). We dried and covered the section with glycerol and sealed the sections with nail polish. Images were taken with EVOS M5000 microscope.

### Gene expression analysis.

Total RNA was isolated from mouse intestine, which was perfused with RNAlater (MilliporeSigma, catalogR0901) for 24 hours at 4°C, and then tissues were homogenized in TRIzol reagent (Ambion, catalog 15596026) with beads using PowerLyzer 24 (Mo Bio Laboratories Inc), then extracted by using RNeasy Mini Kit (QIAGEN, catalog 74106) as per the manufacturer’s instructions. cDNA was synthesized from 1 μg of DNase-treated total RNA using Bio-Rad iScript Reverse Transcription Supermix (1708841), and mRNA levels were quantified by qRT-PCR with Advanced Universal SyBr Green Supermix (Bio-Rad, catalog 725271). All samples were run in technical triplicates, and relative mRNA levels were calculated by using the standard curve methodology and normalized to 36B4. All primers are listed in Supplemental Table 1.

### Western blot analysis.

RAW 264.7 cells and BMDMs were lysed in Pierce RIPA Lysis and Extraction Buffer (Thermo Fisher Scientific, catalog 89900), with freshly added Halt Protease Inhibitor Cocktail (100×) (Thermo Fisher Scientific, catalog 78430). Crude lysates were centrifuged at 14,000*g* for 15 minutes, and protein concentrations were determined using a Bio-Rad protein assay reagent. Samples were diluted in SDS sample buffer. Protein concentrations were measured by bicinchoninic acid assay method. Bound proteins were resolved by SDS-PAGE and transferred to nitrocellulose membranes (Bio-Rad, catalog 170-4159). Individual proteins were detected with specific antibodies (anti-FXR, Santa Cruz Biotechnology catalog sc-13063; anti–β-Actin, CST, catalog 4970s) and visualized on film using horseradish peroxidase–conjugated secondary antibodies (Bethyl Laboratories, Inc A120101P) and Western Lightning enhanced chemiluminescence (PerkinElmer Life Sciences).

### BMDM culture and in vitro polarization.

Bone marrow was collected from tibias and femurs using the aseptic technique. Muscle tissues were trimmed off with scissors, and tibias and femurs were washed with 75% ethanol 3 times, followed by DPBS washes 3 times. The tibias and femurs were cut open from both ends, and the bone marrow was flushed out with cold DPBS with the 20-gauge needles on the 10 mL syringes. After centrifuging at 300*g* at 4°C for 5 minutes, the pellet was treated with red blood cell lysis buffer (VWR) for 5 minutes at room temperature. The bone marrow cells were counted and cultured with DMEM/F12 containing 10% FBS, 20 mM HEPES (Gibco), Penicillin/Streptomycin (PS, Corning), and 20 ng/mL M-CSF (Peprotech) at 37°C and 5% CO_2_ for 5 days for BMDM differentiation. For M1 and M2 macrophage in vitro polarization, BMDMs were treated with 50 ng/mL LPS + 20 ng/mL IFN-γ (M1 macrophage) and 10 ng/mL IL4 + 10 ng/mL IL13 (M2 macrophage) for 24 hours. To assess the effects on M1 and M2 cells, we treated BMDMs with FexD (10 μM) and OCA (1 μM) for the last 6 hours of in vitro polarization. RNA was extracted from M1 and M2 cells, and M1/M2 marker gene expression was measured by qRT-PCR. To examine the effects on M1 cytokines’ stimulation, we treated with FexD (20 μM) and OCA (10 μM) on cells at the same time with M1-polarizing cytokines for 6 hours. Cytokines induced in M1 were measured from cultured supernatant by ELISA (see *Cytokine and cancer tumor marker measurement*).

### In vitro T cell culturing and differentiation.

Naive CD4^+^ T cells isolated by either Miltenyi Biotec or Stemcell Technologies EasyStep Naive CD4^+^ T cell isolation kit (62). A total of 1 × 10^5^ T cells were plated per well in a 96-well plate and cocultured with Dynabeads Mouse T-Activator CD3/CD28 (Gibco). Naive CD4^+^ T cells were in vitro differentiated into Th17 cells in the presence of anti-CD3 (2 μg/mL, Bio X Cell), anti-CD28 (2 μg/mL, Bio X Cell), hTGF-β (2 ng/mL, Peprotech), and IL6 (20 ng/mL, Peprotech). For macrophage/T cell crosstalk assay, after 1 day of Th17 cell differentiation, T cells were subsequently treated with 50 μL of BMDM culture medium in the conditions of M0 (PBS), M1 (LPS + IFN-γ), M2 (IL4+IL13), M1+FexD (20 μM), M1+OCA (10 μM), M2+FexD (20 μM), and M2+OCA (10 μM) for an additional 2 days. IL1β (10 ng/mL) and IL23 (10 ng/mL) were further added as the Th17 cell differentiation positive control. Cells were then incubated with PMA (50 ng/mL, MilliporeSigma) and ionomycin (500 ng/mL, MilliporeSigma) for 1 hour prior to the addition of GolgiPlug (10 μg/mL, BD Biosciences) for an additional 3–4 hours. Cells were then harvested for flow cytometry and qRT-PCR. Cell culture supernatant was collected for ELISA analysis (see *Cytokine and cancer tumor marker measurement*).

### Intestinal immune cell isolation.

Mouse small intestines and colons, devoid of fat tissue, were washed in ice-cold RPMI 1640 (Thermo Fisher Scientific), and identified Peyer’s patches were eliminated. Small intestines were longitudinally transected, washed twice in ice-cold RPMI 1640 to remove luminal contents, and then dissected into 1 cm pieces and vigorously shaken in ice-cold RPMI 1640 to remove the mucus. Epithelial cells were dissociated during two 15-minute incubations on a shaker at 37°C in RPMI 1640, 100 U/mL penicillin and 100 μg/mL streptomycin (Corning), 5% FBS (Corning), 5 mM EDTA, 20 mM HEPES, and 1 mM DTT (freshly added), with dissociated cells collected by filtration (100 μm filter) (Thermo Fisher Scientific) after each step. Residual tissue was digested with 0.1 mg/mL Liberase TL (Roche, freshly added) in RPMI 1640, 1% PS, 20 mM HEPES, and 20 μg/mL DNase I (MilliporeSigma, freshly added) for 40 minutes on a shaker at 37°C. Lamina propria cells were further enriched using a discontinuous 44% over 67% Percoll gradient. Mesenteric lymphocytes and splenocytes were harvested by mechanical disruption on 100 μm nylon mesh filters (Thermo Fisher Scientific). In splenocyte preparations, residual red blood cells were lysed in ACK (8 minutes of incubation at room temperature in ammonium-chloride-potassium lysing buffer). Mesenteric lymphocytes were further dissociated with 33 μg/mL Liberase TL (Roche) and 100 μg/mL DNase I in DMEM for 15 minutes at 37°C. To evaluate cytokine production, isolated cells were treated with 50 ng/mL PMA and 500 ng/mL ionomycin for 4–6 hours in the presence of 10 μg/mL GolgiPlug.

### Flow cytometry.

Mouse lamina propria immune cells were labeled with indicated antibodies in PBS containing 2% FBS. Immune cells were initially blocked with FC block (BD, catalog 553142, 30 minutes on ice). Cell surface lineage markers included APC-Cy7 Anti-Mouse CD3 (BioLegend, catalog 100330), BV605 Anti-Mouse CD8a (BioLegend, catalog 100743), Alexa Fluor 700 Anti-Mouse CD45.2 (BioLegend, catalog 109822), BV510 Anti-Mouse CD4 (BioLegend, catalog 100553), PE Anti-Mouse TCRβ (Life Technologies, catalog 12-5961-82), Zombie UV (BioLegend, catalog 423107), BV785 Anti-Mouse CD11c (BioLegend, catalog 117335), BV605 Anti-Mouse Ly6C (BioLegend catalog 128035), BV421 Anti-Mouse F4/80 (BioLegend, catalog 123131), BV510 Anti-Mouse CD11b (BioLegend, catalog 101245), PE Anti-Mouse CX3CR1 (BioLegend, catalog 149006), PE-Cy7 Anti-Mouse Ly6G (BioLegend, catalog 127618), PerCP-Cy5.5 Anti-Mouse CD206 (BioLegend, catalog 141715), FITC AF488 Anti-Mouse CD68 (BioLegend, catalog 137005), APC-Cy7 anti-Mouse MHCII (BioLegend, catalog 107628), and APC AF647 Anti-Mouse CD64 (BioLegend, catalog 139306). Intracellular staining was performed after fixation with the Foxp3/Transcription Factor Staining Kit (eBioscience, catalog 005523) and incubated with indicated antibodies. Intracellular antibodies include BV421 Anti-Mouse RORγt (BD Horizon, BDB562894), PE-Cy7 Anti-Mouse IL17A (BioLegend, catalog 506921), PerCP-Cy5.5 Anti-Mouse IFNγ (BioLegend, catalog 505821), and APC AF647 Anti-Mouse IL23 (BD Biosciences, catalog 565317). Detailed sorting strategies for gut macrophages and T cells are presented in Supplemental Figures 10 and 11. All information of antibodies is listed in Supplemental Table 2. The staining panels were analyzed on 5-laser, 18-color, custom-configuration BD LSRFortessa and/or sorted on 5-laser, 18-color custom-configuration FACSAria III. All data collection was performed with FACSAria II cell sorters (BD Biosciences) at UWCCC and Salk’s Flow Cytometry Core and analyzed with FlowJo software (Tree Star).

### Ex vivo gut macrophage culture.

Enriched F4/80^+^ gut macrophages (Stemcell Technologies) were cultured in DMEM/F12 containing 10% FBS, 20 mM HEPES (Gibco), PS (Corning), and 40 ng/mL M-CSF (Peprotech) and GM-CSF at 37°C and 5% CO_2_ for 12 hours. Gut macrophages were then treated with FexD or OCA for various time points prior to qRT-PCR, ELISA, or FACS analysis.

### Bone marrow transplantation.

Donor mice (WT C57BL/6 or *FXR*-KO B6, both CD45.2^+^) were sacrificed and bone marrow cells (BMCs) harvested from the tibias and femurs. BMCs were suspended in saline containing 2% heat-inactivated FBS. Recipient mice (WT C57BL/6J, CD45.1^+^) were given 8.5–11 Gy whole-body lethal irradiation from an x-ray or gamma irradiation source. One day after the last irradiation, recipient mice were intravenously injected with 150 μL volume, 1 × 10^7^ BMCs from donors via the retro-orbital vein using a 27-gauge needle. After 4 weeks, blood was collected from recipient mice to check reconstitution effectiveness by staining CD45.2 and CD45.1. After 8 weeks for further reconstitution in the intestinal tissue, recipient mice were harvested for tissues/cells.

### Bioinformatic analysis.

To compare the expression of FXR target genes and genes encoding macrophage cytokines between clinical adjacent normal tissues and tumors, FPKM-normalized RNA-Seq data extracted from TCGA COAD and READ projects, along with biospecimens’ information was used. The CAC data sets are listed in Supplemental Table 3. To evaluate the differential expression of FXR target genes and genes encoding macrophage cytokines and surface markers between inflammatory tissues and normal controls, batch-corrected and normalized data of the IBD combined cohort were directly downloaded from GitHub IBD TaMMA repository and analyzed. Data extracted from 3 representative individual National Center for Biotechnology Information (NCBI) Gene Expression Omnibus (GEO) cohorts were also used for analysis. The box plots for above analyses were generated by R package *ggplot2* with *P* value calculated using Wilcoxon’s test. To assess the correlation between *Fxr* and macrophage-related genes’ expression, the raw RNA-Seq data of mouse intestinal macrophages were extracted from GEO (GSE140788) and reanalyzed. Briefly, all read files were downloaded from NCBI SRA database and dumped into fastq files. The single-end reads were mapped to GRCm39 using splice-aware STAR aligner (version 2.7.1a). The uniquely mapped reads were counted using HTSeq and normalized as reads per kilobase million, to be consistent with the previous analysis. The scatterplots were generated by R package *ggscatter*, with correlation coefficients and *P* values computed using Spearman. For survival analysis, the RNA-Seq data from TCGA COAD and READ projects were scaled as *z* score value for individual genes, and patient survival data were combined. Data from patients with a stage I diagnosis while enrolled were refined to minimize the effects on M2 macrophages. R package *survival* was used for plotting survival curves. The log-rank *P* value and HR were computed from the Cox proportional hazards model. The version of base R for all the packages used is 4.1.2.

### Statistics.

Statistical analyses were performed with Prism 9.0 (GraphPad Inc.) and R software (www.r-project.org). Student’s 2-tailed unpaired *t* test or 1-way or 2-way ANOVA tests were applied to compare 2-group or multiple-group variables followed by Bonferroni’s, Tukey’s, or Fisher’s multiple comparisons tests. Student’s 2-tailed *t* test was applied to compare continuous variables, and Pearson’s correlation coefficient was used for testing linearity degree. Survival curves were plotted with the Kaplan-Meier method and compared by log-rank tests. Data are presented as means of at least 3 independent replicates ± SEM, and *P* < 0.05 was considered statistically significant. More detailed materials and methods can be found in the figure legend information.

### Study approval.

All animal experiments were performed in the specific pathogen–free facilities at the University of Wisconsin–Madison following the Institutional Animal Care and Use Committee’s guidelines and with the Committee’s approval.

### Data availability.

The RNA-Seq data used in this paper have been all published previously and deposited in NCBI’s SRA, accession numbers GSE140788, GSE165512, GSE117993, and GSE109142. The data that support the findings of this study are available from the corresponding author upon request. Values for all data points in graphs are reported in the Supporting Data Values file.

## Author contributions

TF designed and supervised the research. TF initiated the project and completed animal experiments presented in Figures 1–4 and Supplemental Figures 1–4 and 7. XD continued the project and completed the rest together with TF. XD and TF performed the majority of all the experiments and analyzed results, with experimental help from MQ and FS and with technical consulting from YL and YZ. CC analyzed genomic data. SC and CL helped with the BA composition analyses. NVU, WX, PM, RH, and SL provided scientific input. XD and TF prepared the manuscript. Based on the actual data contribution, XD and TF shared co–first authorship.

## Supplementary Material

Supplemental data

Supporting data values

## Version 1 01/23/2024

Electronic publication
