# Supplementary material for: Farnesoid X receptor mediates macrophage-intrinsic responses to suppress colitis-induced colon cancer progression
Source: JCI Insight. 2024 Jan 23;9(2):e170428. doi: 10.1172/jci.insight.170428 (PMC10906220; doi:10.1172/jci.insight.170428)
Supplement: Supplemental data [file jciinsight-9-170428-s152.pdf]

Supplementary Figure 1

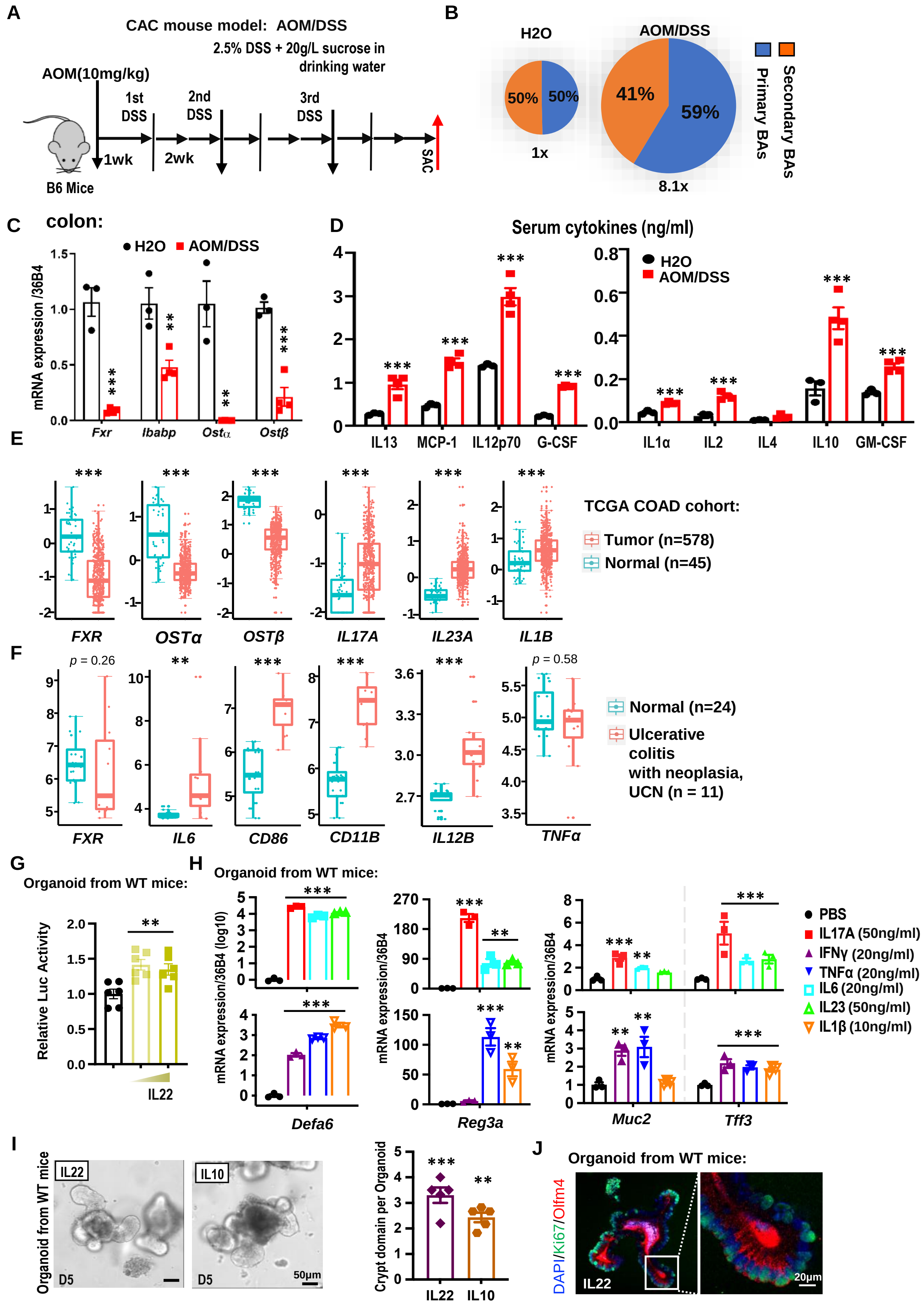

Supplementary Figure.1 | inflammation aggravated in the CAC model promotes intestinal organoid growth.

(A) Schematic of CAC (AOM/DSS) mouse model: WT mice are injected with AOM once, then treated with 2.5% DSS chronically for 1 week and rest for 2 weeks and repeated 3 times for tumor generation.

(B) Primary and secondary BAs proportion in CAC mouse model.

(C) Expression of *Fxr* and its target genes (*Ibabp, Ostα, Ostβ*) in CAC mouse model.

(D) Serum cytokine levels measured by ELISA in 18-week-old CAC mouse models (n= 3-5). The experimental scheme is described in **Figure 1A**.

(E) Relative expression (FPKM values) of presented genes based on RNA-seq data of normal and tumor tissue samples from combined Cancer Genome Atlas (TCGA) CRC cohorts.

(F) Relative expression (FPKM values) of presented genes based on RNA-seq data of normal and UC with neoplasia samples.

The below experimental scheme is described in **Figure 1G-K**.

(G) Proliferation of organoids derived from WT mice in response to increasing cytokines concentrations IL22 (10, 20 ng/ml), measured by CellTiter-Glo Luminescent 3D Cell Viability Assay.

(H) Paneth cell marker genes (*Defa6*, *Reg3g*), Goblet cell marker genes (*Muc2, Tff3),* expression in organoids from **(G)** treated with pro-inflammatory cytokines.

(I) Brightfield images of branching WT organoids, treated on day 2 for 48hrs (images were taken after 24hrs) with IL22 (20 ng/ml) and IL10 (20 ng/ml). Scale bar 50μm. Branching of the above organoids was quantified in the crypt domain per organoid from 5 individual organoids.

(J) Co-immunostaining images of WT organoids treated with IL22 (20 ng/ml). Stem cell gene marker Olfm4 is in red; proliferating gene marker Ki67 is in green, the nucleus is counterstained with DAPI in blue. Circled with higher magnification is presented in the bottom panel. Scale bar 20μm.

Experiments were independently replicated twice, and representative data are shown as the mean ± SEM. P-values are determined with Student's unpaired t-test and one-way ANOVA test followed by Tukey's multiple comparisons. * $p<0.05$; ** $p<0.01$; *** $p<0.005$.

Supplementary Figure 2

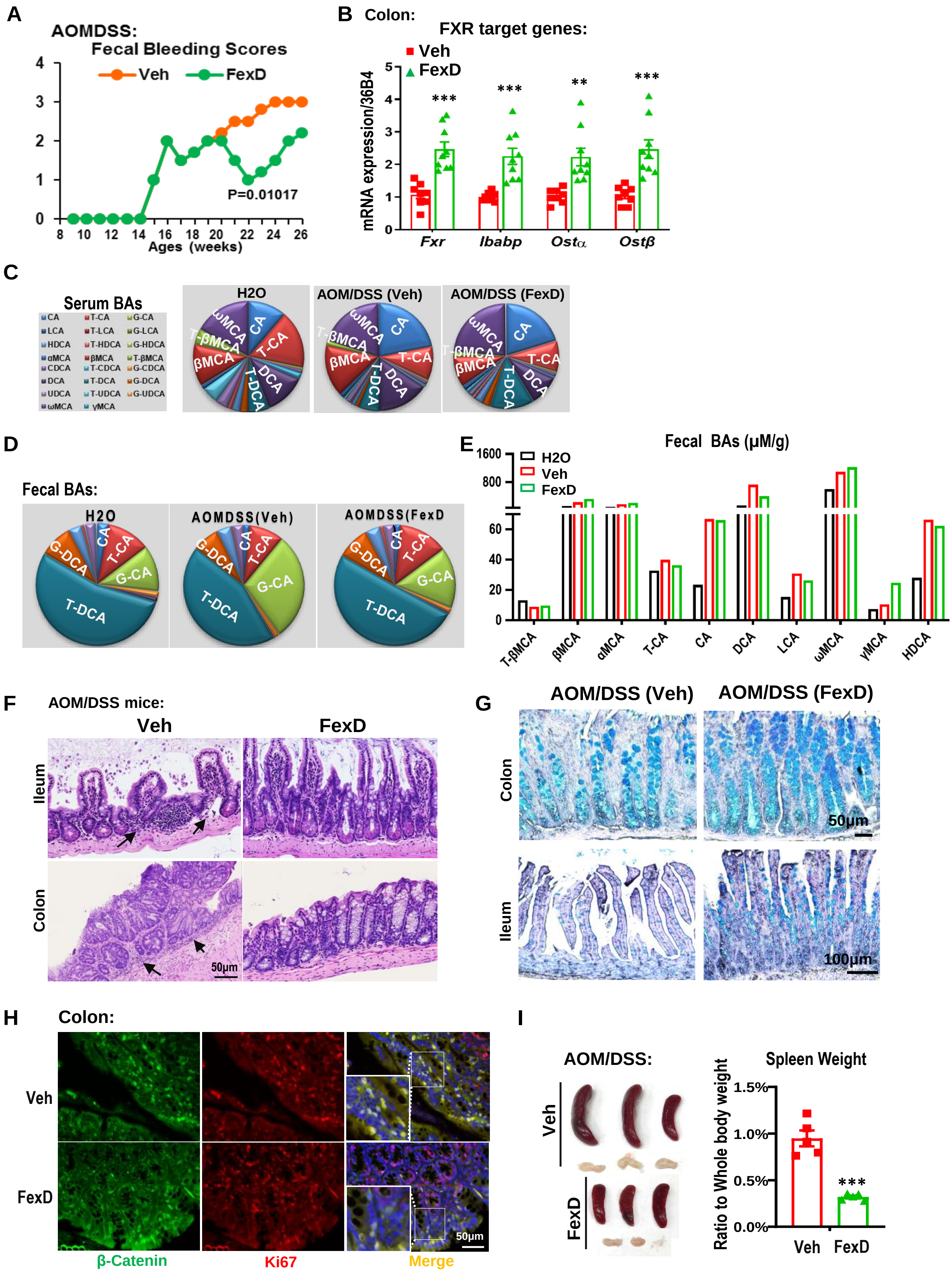

Supplementary Figure.2 | FXR protects against chronic DSS-induced colon cancer.

The experimental scheme is described in **Figure 2A**.

(A) Progressive changes in fecal bleeding scores measured by fecal occult blood test in above treatment groups.

(B) Serum BA composition shifted by FexD in CAC mice models.

(C) Expression of *Fxr* and its target genes (*Ibabp, Ostα, Ostβ*) in CAC mouse model under FexD treatment.

(D-E) Fecal BA levels **(D)** and composition **(E)** shifted by FexD in CAC mice models. (data represents fecal samples pooled from 5 mice (one cage), n=10).

(F) H&E staining of ileum and colon part in above treatment groups, scale bar 50μm.

(G) PAS and Alcian blue staining of the colon (Scale bar 50μm) and ileum (Scale bar 100μm). Segment. Mucosal Goblet cells are stained in magenta color.

(H) Co-immunostaining images of β-Catenin (green) and Ki67 (red) in the tumors on the colon segment in CAC mice, treated with FexD or Vehicle. Scale bar 50μm.

(I) Spleen images and weight of mice in above treatment groups.

Experiments were independently replicated twice, and representative data are shown as the mean ± SEM. * $p<0.05$; ** $p<0.01$; *** $p<0.005$, Student’s unpaired t-test.

Supplementary Figure 3

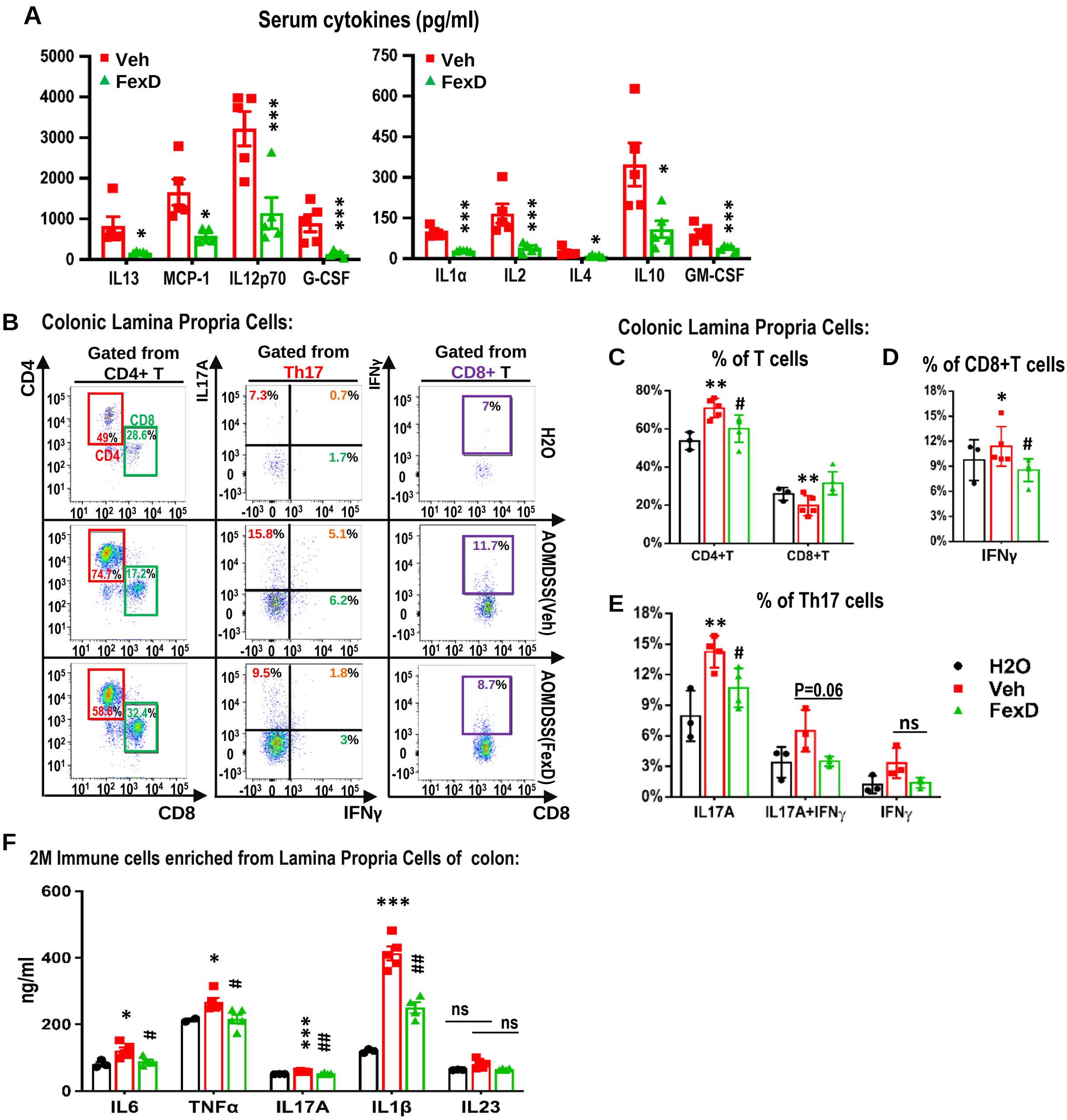

Supplementary Figure.3 | Activation of FXR reduces intestinal inflammation in CAC.

The experimental scheme is described in **Figure 2A**.

(A) Serum cytokine levels measured by ELISA in CAC mice with FexD or Vehicle (n= 3-5).

(B) Representative flow cytometry analyses of the Th17 and IL17A expressing cell populations in the Lamina Propria of the colon with the vehicle and FexD-treated AOM/DSS mice as experimental scheme described in Figure 2A.

(C) The quantification of CD4+ and CD8+ T cell numbers (n=3-5).

(D-E) The quantification of IFNγ and IL17A secreting cells in CD8+ **(D)** and CD4+ **(E)** T cells (n=3-5).

(F) Pro-inflammatory cytokines (IL6, TNFα, IL17A, IL1β and IL23) of 2 million cells isolated from colonic Lamina Propria in CAC mice with FexD or Vehicle (n= 3-5), measured by ELISA.

Experiments were independently replicated twice, and representative data are shown as the mean ± SEM. P-values are computed with Student's unpaired t-test and one-way ANOVA test followed by Tukey's multiple comparisons. * DSS versus water, $^{\#}$ FexD versus Vehicle in AOMDSS cohort; *, $^{\#}$ $p<0.05$; **, $^{\#\#}$ $p<0.01$; ***, $^{\#\#\#}$ $p<0.005$.

Supplementary Figure 4

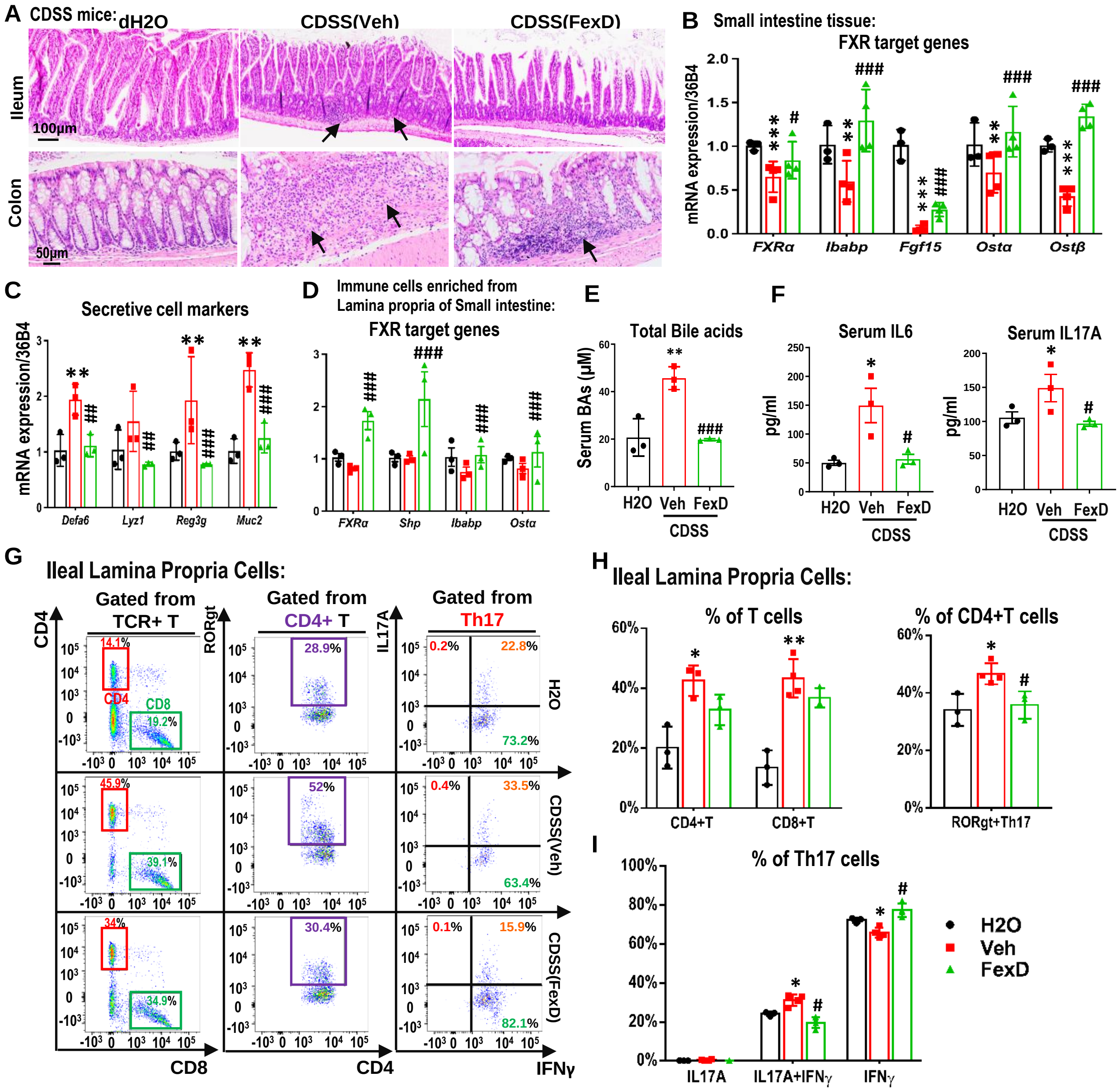

Supplementary Figure.4 | FXR activity in Macrophages attenuates DSS-induced intestinal inflammation.

Panel **A-G**: Additional analyses of WT mice under CDSS described in **Fig. 4B**.

(A) H&E staining of the small intestine, arrows indicate neutrophil infiltration. Scale bar 100μm.

(B) Expression of FXR target genes in the small intestine, measured by qRT-PCR.

(C) Expression of secretive cell marker genes in small intestine tissue, measured by qRT-PCR.

(D) Expression of FXR target genes of the immune cells isolated from Lamina Propria of the small intestine, measured by qRT-PCR.

(E-F) Total BAs **(E)**, IL6, and IL17A **(F)** in the serum.

(G-I) Representative flow cytometry analysis of the percentage of CD4+ and CD8+ T cells, Th17 cells, and $IL17A^+$ and $IFN\gamma^+$ pathogenic Th17 cells out of parental cells **(G)**, with quantification presented in **H-I** (n=3-4).

Experiments were independently replicated twice, and representative data are shown as the mean ± SEM. P-values are computed with Student's unpaired t-test and one-way ANOVA test followed by Tukey's multiple comparisons. * DSS versus water, $^{\#}$ FexD versus Vehicle in AOMDSS cohort; *, $^{\#}$ $p<0.05$; **, $^{\#\#}$ $p<0.01$; ***, $^{\#\#\#}$ $p<0.005$.

Supplementary Figure 5

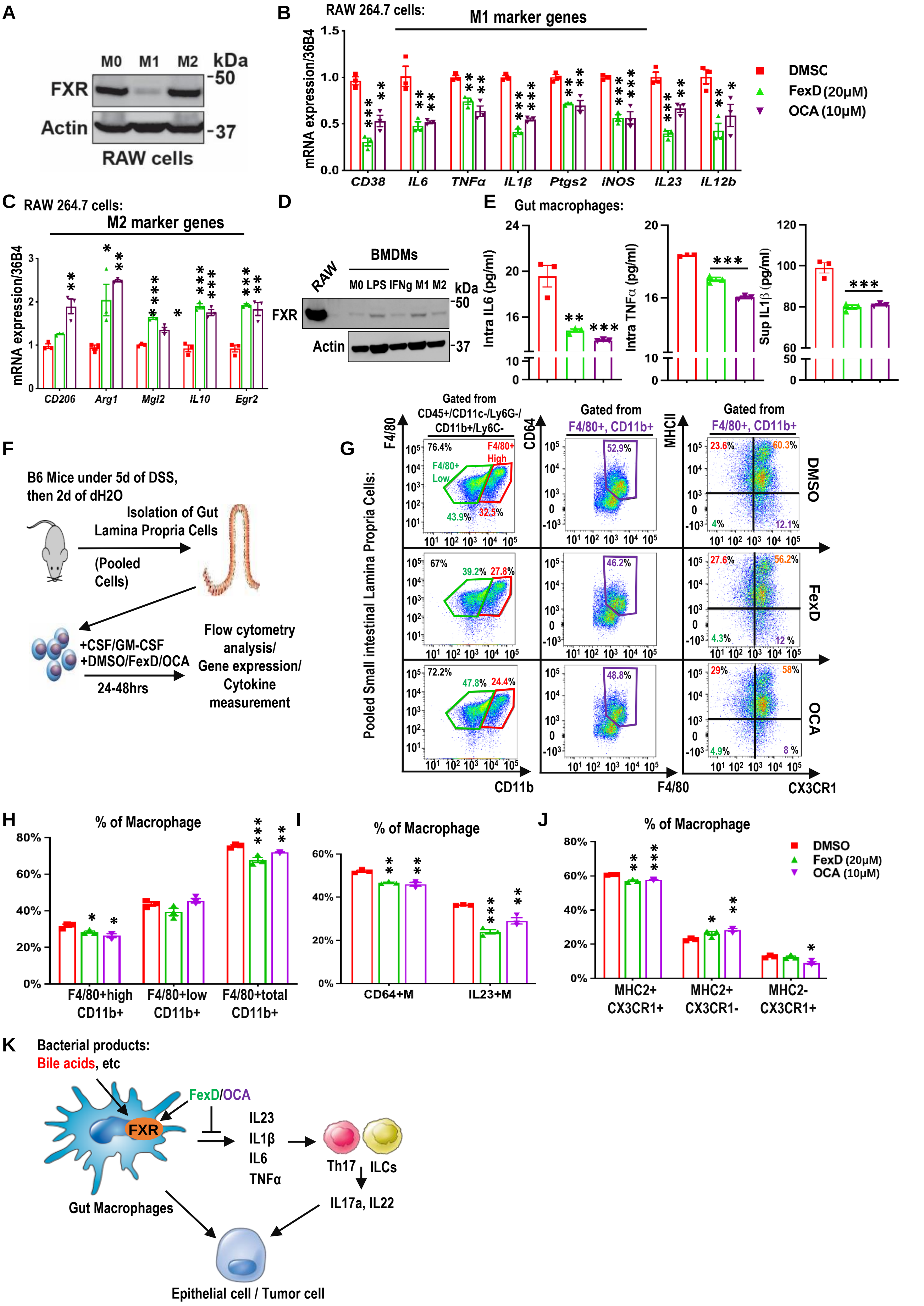

Supplementary Figure.5 | FXR modulates macrophage function *in vitro*.

(A) Expression of FXR protein level in M1 and M2 types of RAW264.7 cells, measured by Western blot with actin as a loading control.

(B-C) Expression of M1 marker genes **(B)** and M2 marker genes **(C)** in RAW264.7 cells with FexD, OCA, or Vehicle, measured by qRT-PCR (n=3).

(D) Expression of FXR protein level in M1 and M2 types of BMDM cells, measured by Western blot with actin as a loading control.

The below experimental scheme is described in **Figure 6C**.

(E) Gut macrophages intracellular synthesized cytokines (IL6, TNFα) and secreted cytokines (IL1β) were measured by ELISA (n=3).

(F) Experimental scheme of intestinal Lamina isolated from WT B6 mice under 5 days of DSS and 2 days of recovery time, then subjected to FXR agonists treatment for 24hr. Supernatant and cell samples were harvested for examination for below panel **G-J.**

(G-J) Representative flow cytometry analysis of small intestinal Lamina Propria CD11b+high, F4/80+; CD11b+low, F4/80+; CD11b+, F4/80+ total macrophages, and CD68+, CD64+ CD206+, IL23+ macrophage; and CX3CR1+, MHCII+; CX3CR1-, MHCII +; and CX3CR1+, MHCII-macrophage **G**. Data quantification is presented in **H-J.**

(K) Working scheme of macrophages sense bile acids, activate pro-inflammatory responses, cross-talk to potential Th17 and ILC3 cells, together contribute to tumorigenesis.

Experiments were independently replicated three times, and representative data are shown as the mean ± SEM. P-values are computed with Student's unpaired t-test and one-way ANOVA test followed by Tukey's multiple comparisons. * DSS versus water; * $p<0.05$; ** $p<0.01$; *** $p<0.005$.

Supplementary Figure 6

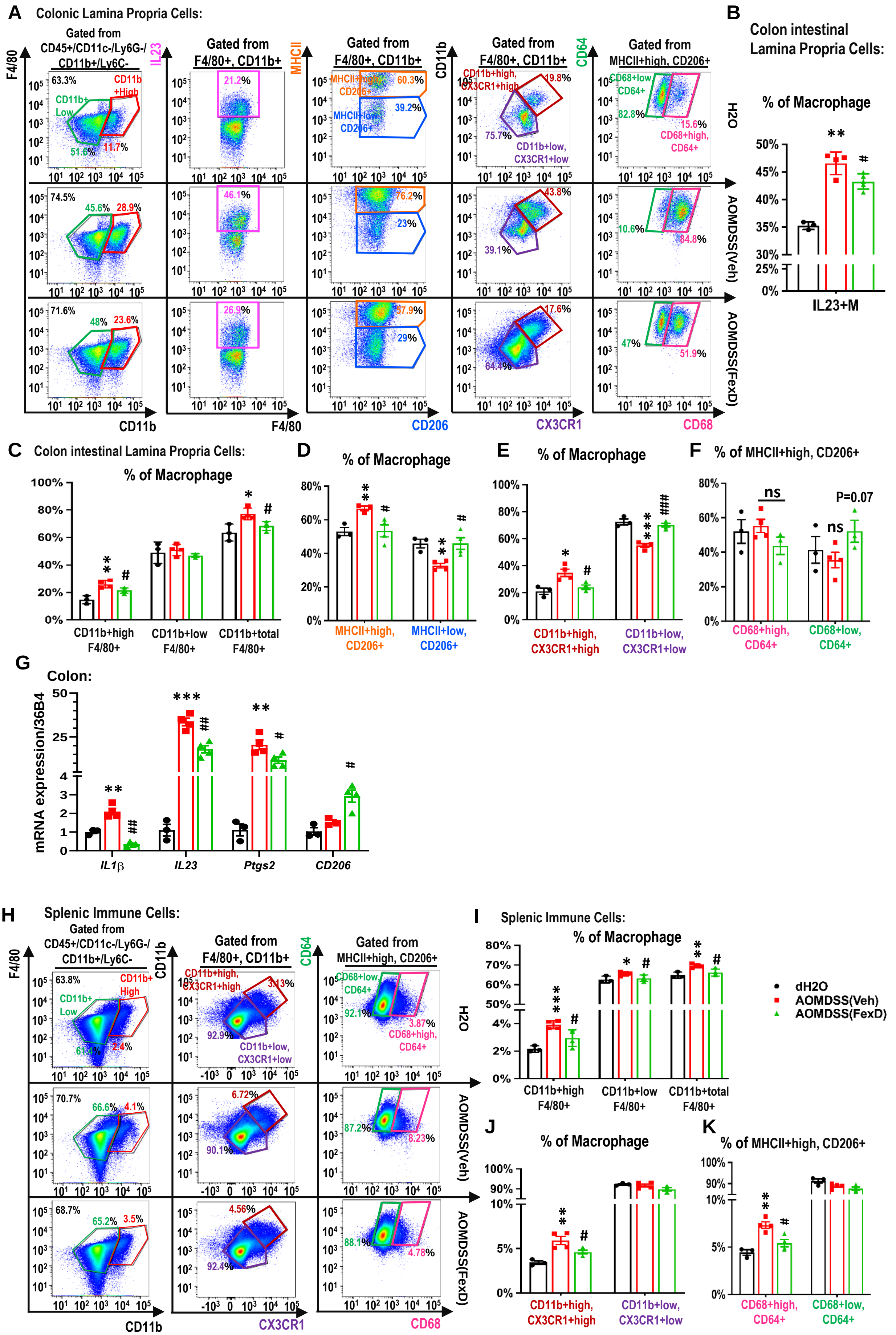

Supplementary Figure.6 | FXR modulates colonic and splenic macrophage function *in vivo*.

All panels are referred to the experimental scheme described in **Figure 7A.**

(A-F) Representative flow cytometry analysis of colonic Lamina Propria CD11b+high, F4/80+; CD11b+low,F4/80+; CD11b+,F4/80+ total macrophages, and IL23+ macrophage; MHCII+high,CD206+; MHCII+low,CD206+; CD11b+high,CX3CR1+high; CD11b+low,CX3CR1+low; CD68+low,CD64+; CD68+high,CD64+ macrophage **(A)**. Data quantification is presented in **B-F.**

(G) Gene expression of M1-like and M2-like gut macrophage markers in colon.

(H-J) Representative flow cytometry analysis of colonic Lamina Propria CD11b+high, F4/80+; CD11b+low,F4/80+; CD11b+,F4/80+ total macrophages, and IL23+ macrophage; MHCII+high,CD206+; MHCII+low,CD206+; CD11b+high,CX3CR1+high; CD11b+low,CX3CR1+low; CD68+low,CD64+; CD68+high,CD64+ macrophage **(H)**. Data quantification is presented in **I-J.**

Experiments were independently replicated twice, and representative data are shown as the mean ± SEM. * DSS versus water, # FexD versus Vehicle; *, $^{\#}$ $p<0.05$; **, $^{\#\#}$ $p<0.01$; ***, $^{\#\#\#}$ $p<0.005$, ns --not significant, Student's unpaired t-test.

Supplementary Figure 7

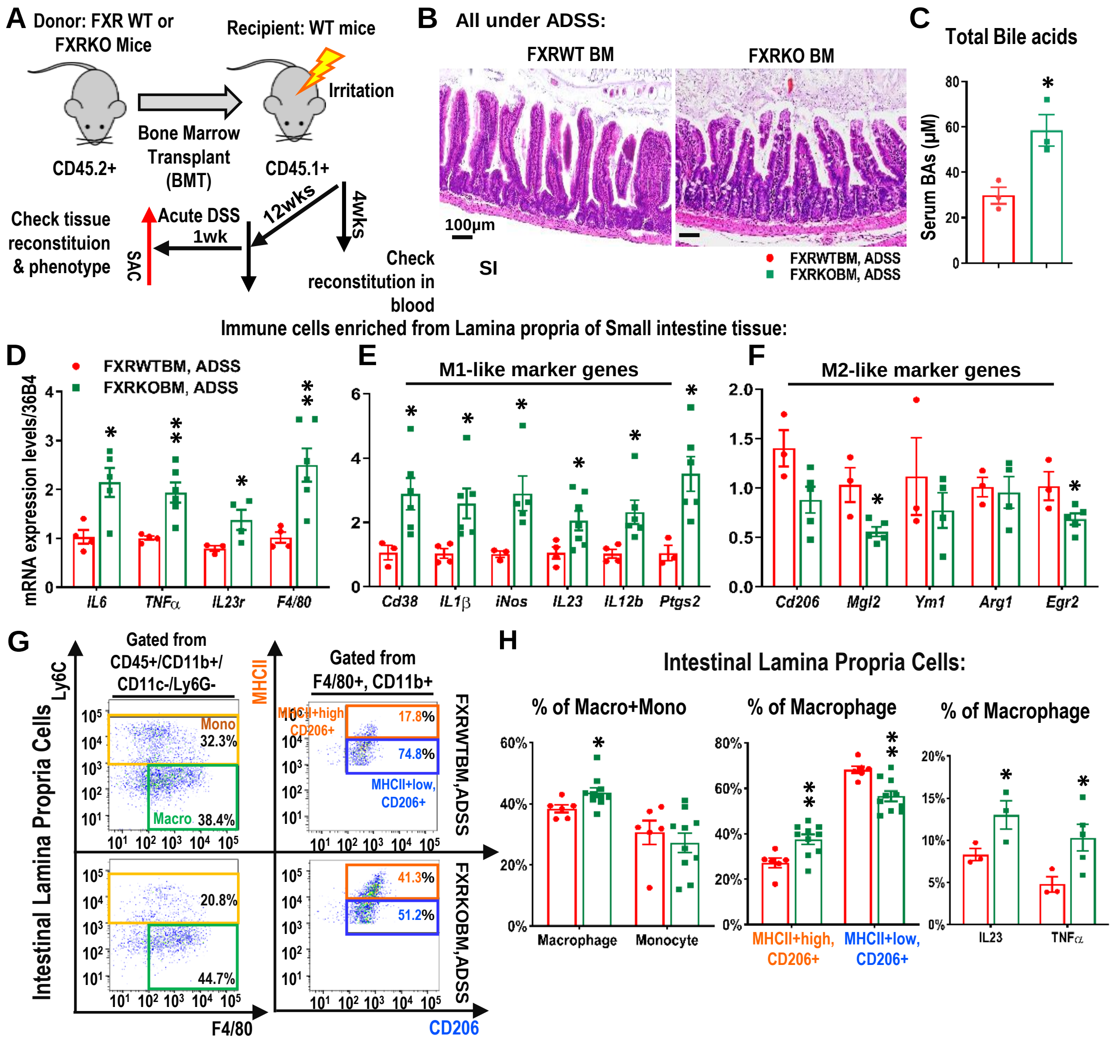

Supplementary Figure.7 | FXR activity in immune cells attenuated DSS-induced damage.

(A) Scheme of Bone Marrow Transplants from WT (*FXR*WTBM) and FXRKO (*FXR*KOBM) donor mice to irradiated WT recipient mice prior to ADSS challenge.

(B) Representative H&E staining of small intestine (scale bar 100 μm).

(C) Serum bile acid levels (*FXR*WTBM group, n=3; *FXR*KOBM group, n=4).

(D-F) Expression of general pro-inflammatory cytokines and macrophage genes, M1-like marker genes and M2-like marker genes in the immune cells isolated from the Lamina Propria of the small intestine, measured by quantitative RT-PCR (n=3-6 per arm).

(G) Representative flow cytometry analysis of monocytes and macrophages, macrophage subtypes, in viable CD45+Ly6G-CD11c-CD11b+ immune cells.

(H) Quantification of macrophages and monocytes in parental immune cells; MHCII+high, CD206+ and MHCII+low, CD206+ in macrophages; IL23 and TNFα secreted by macrophages (n=3-6 per *FXR*WTBM arm, n=3-10 per *FXR*KOBM arm). **FXR*WTBM group compared to *FXR*KOBM group. Data represents mean ± SEM. * and #, p<0.05; ** and ##, p<0.01; *** and ###, p<0.005, Student's t-test (unpaired). These experiments were independently repeated twice with similar outcomes.

Supplementary Figure 8

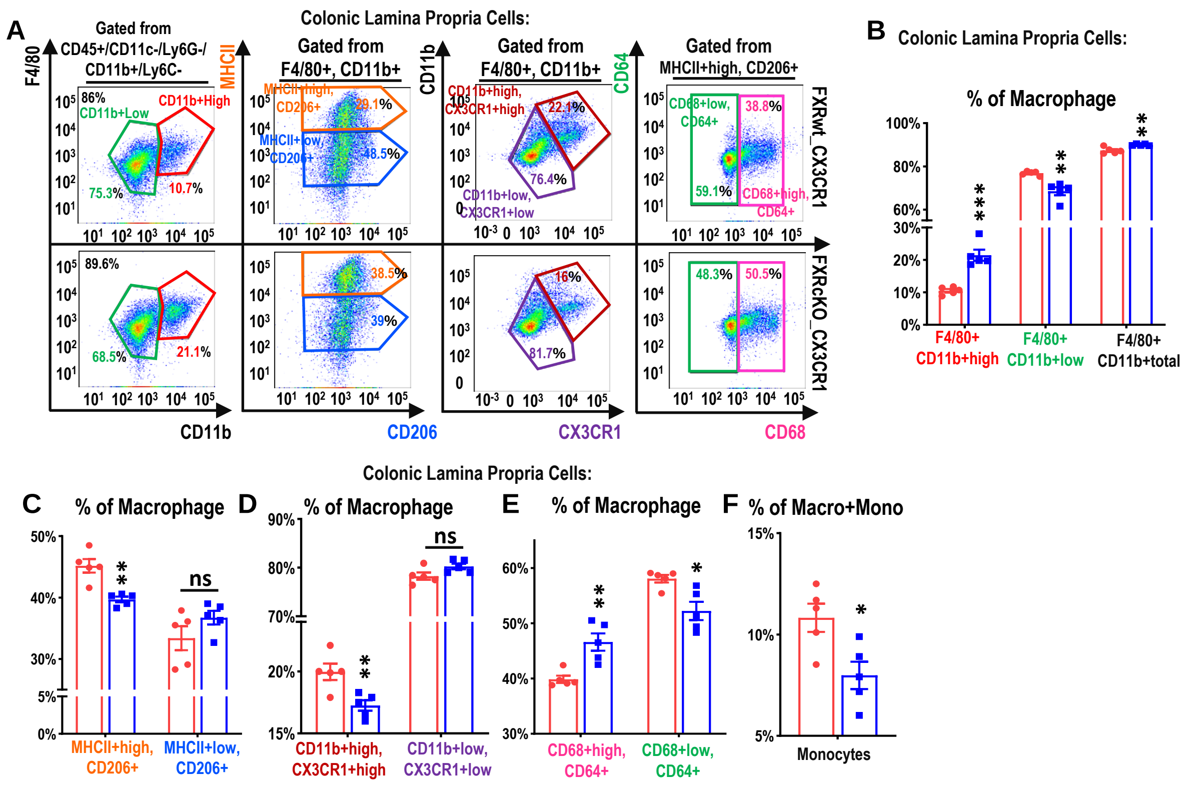

Supplementary Figure 8. FXR attenuates colonic gut macrophages responses to inflammation.

All panels are referred to the experimental scheme described in **Figure 8A.**

(A) Representative flow cytometry analyses of colonic Lamina Propria cells: CD11b+high, F4/80+; CD11b+low, F4/80+; CD11b+, F4/80+ total macrophages, and MHCII+high, CD206+; MHCII+low, CD206+; CD11b+high, CX3CR1+high; CD11b+low, CX3CR1+low; CD68+low, CD64+; CD68+high, CD64+ macrophages.

(B-F) Data Quantification of macrophage, subtypes of macrophages, and monocytes corresponding to above flow analysis. Experiments were independently replicated 2 times, and representative data are shown as the mean ± SEM. * *FXR*wt_*CX3CR1* versus *FXR*cKO_*CX3CR1*. *, $p<0.05$; **, $p<0.01$; ***, $p<0.005$, Student's t-test (unpaired).

Supplementary Figure 9

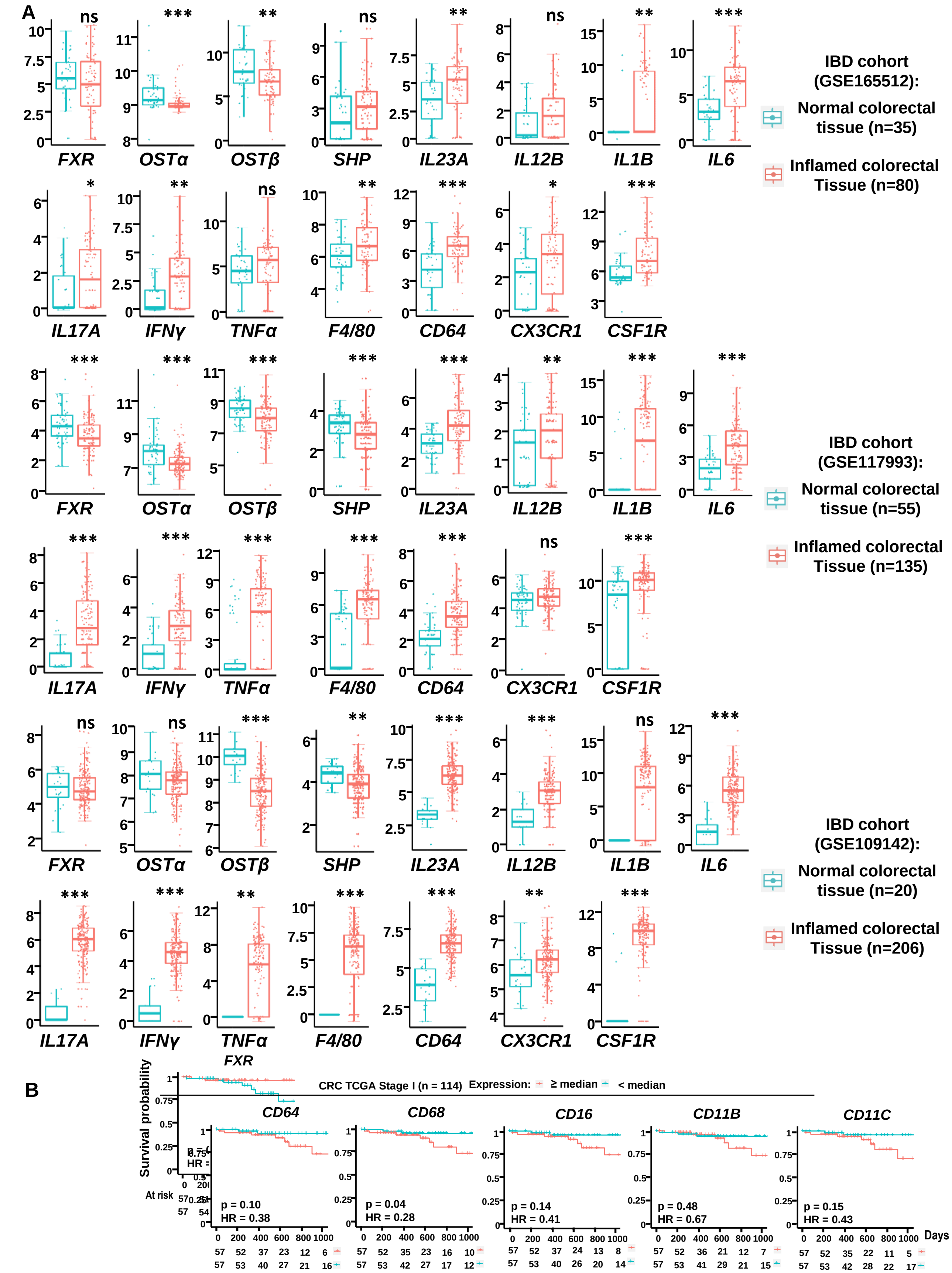

Supplementary Figure 9. The association of gene expression of FXR pathway genes, pro-inflammatory cytokine genes and macrophage signature genes.

(A) Relative expression levels of presented genes based on the RNA-seq data of normal and inflamed colorectal tissues from different IBD cohorts (GSE165512; GSE117993; GSE109142), sample numbers are indicated in figure and expression presented as FPKM values. Wilcoxon-test, * p<0.05; ** p<0.01; *** p<0.005, ns --not significant.

(B) Survival curves (log-rank test) for FXR and macrophage signature gene from CRC TCGA stage I patients.

Supplementary Figure 10 Macrophage analysis panel

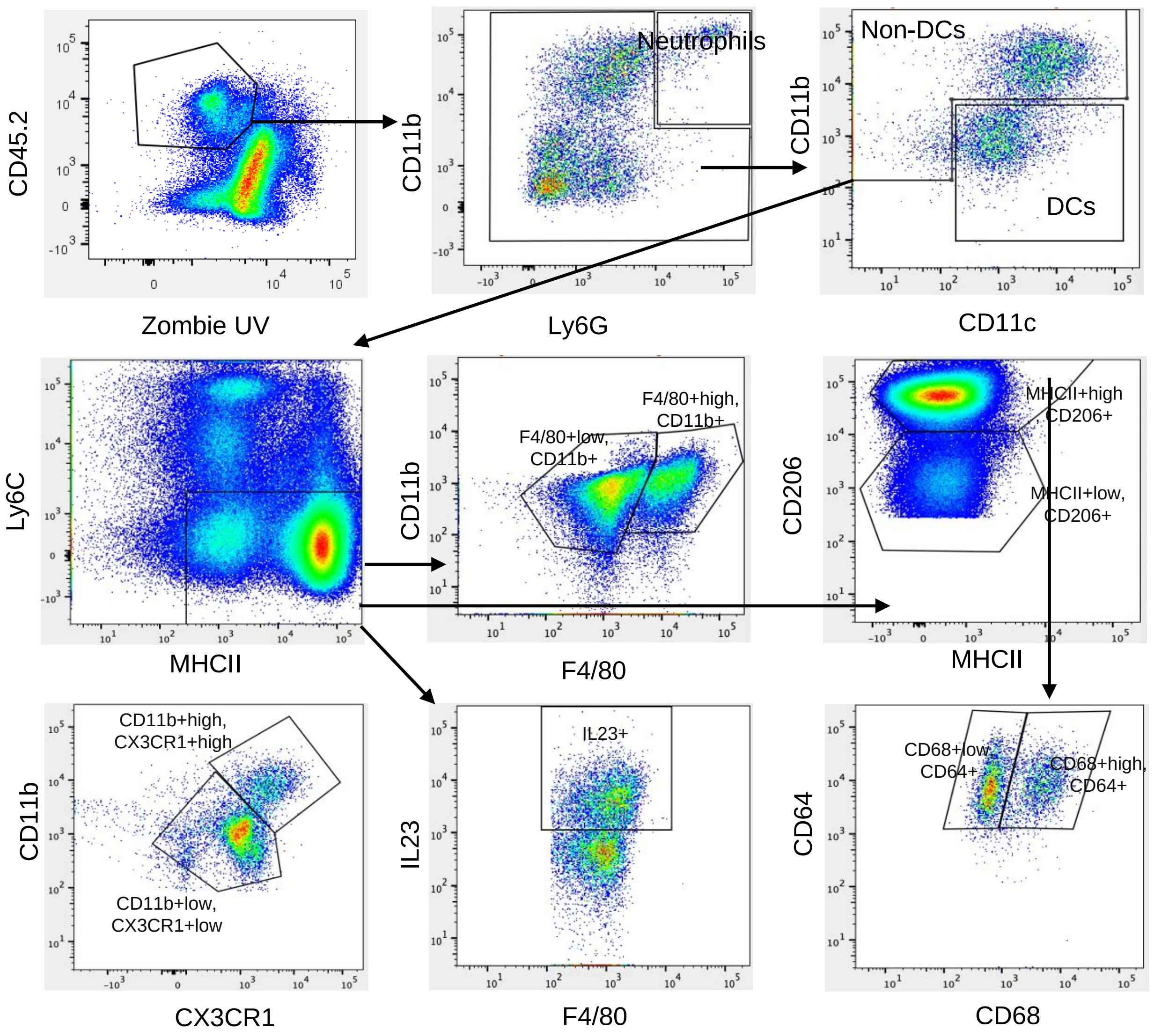

Gating strategy for macrophage subpopulations.

Supplementary Figure 11 Conventional T cell panel

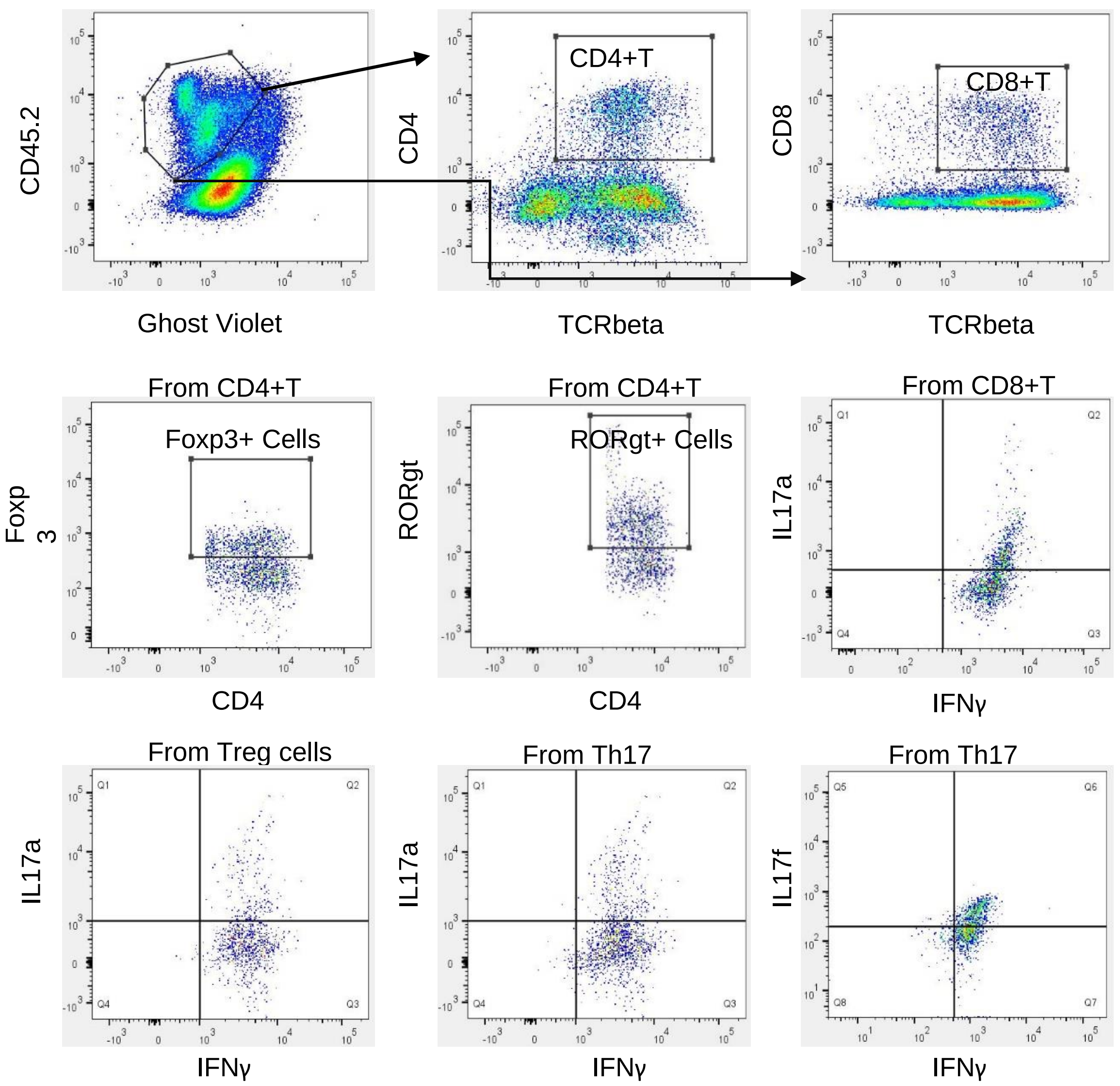

Gating strategy for conventional T cells.
Cytokines in each population are gated similarly to IL17A.

Supplementary Table 1. RT-qPCR primer list.

Primer Name	Sequence (5 to 3)
m36b4-F	ACCTCCTTCTTCTTCCAGGCTTT
m36b4-R	CCCACCTTGTCTCCAGTCTTT
mFXRα-F	GGCCTCTGGGTACCACTACA
mFXRα-R	ACATCCCCATCTCTTTGCAC
mFgf15-F	GCCATCAAGGACGTCAGCA
mFgf15-R	CTTCCTCCGAGTAGCGAATCA
mIbabp-F	CACCATTGGCAAAGAATGTG
mIbabp-R	AACTTGTCACCCACGACCTC
mOSTα-F	TACAAGAACACCCTTTGCCC
mOSTα-R	AGGAATCCAGAGACCAAAGC
mOSTβ-F	GTATTTTCGTGCAGAAGATGC
mOSTβ-R	ATTTCTGTTTGCCAGGATGCT
mShp-F	CTACCCTCAAGAACATTCCAGG
mShp-R	CACCAGACTCCATTCCACG
mLgr5-F	CAAGCCATGACCTTGGCCCTG
mLgr5-R	TTTCCCAGGGAGTGGATTCTATT
mOlfm4-F	CAGCCACTTTCCAATTTCACTG
mOlfm4-R	GCTGGACATACTCCTTCACCTTA
mTnfrsf19-F	ATTCTCTTCCTACTCCACCTG
mTnfrsf19-R	CATAGCCGAAGCCACATTC
mAscl2-F	TAGTGCAGCCTGACCAAATG
mAscl2-R	AAGTCCTGATGCTGCAACGT
mDefa6-F	CCTTCCAGGTCCAGGCTGAT
mDefa6-R	TGAGAAGTGGTCATCAGGCAC
mReg3a-F	GGGCTGAGGGATCCTTCGT
mReg3a-R	AGCCCAATCCAGACGTATGAGT
mReg3g-F	TTCCTGTCCTCCATGATCAAAA
mReg3g-R	CATCCACCTCTGTTGGGTTCA
mMuc2-F	ATGCCCACCTCCTCAAAGAC
mMuc2-R	GTAGTTTCCGTTGGAACAGTGAA
mTff3-F	TTGCTGGGTCCTCTGGGATAG
mTff3-R	TACACTGCTCCGATGTGACAG
mLyz1-F	GGAATGGATGGCTACCGTGG
mLyz1-R	CATGCCACCCATGCTCGAAT
mIL6-F	TAGTCCTTCCTACCCCAATTTCC
mIL6-R	TTGGTCCTTAGCCACTCCTTC
mTNFα-F	CCCTCACACTCAGATCATCTTCT
mTNFα-R	GCTACGACGTGGGCTACAG
mIFNγ-F	ATGAACGCTACACACTGCATC
mIFNγ-R	CCATCCTTTTGCCAGTTCCTC
mIL17a-F	TTTAACTCCCTTGGCGCAAAA
mIL17a-R	CTTTCCCTCCGCATTGACAC

mIl17f-F	TGCTACTGTTGATGTTGGGAC
mIl17f-R	AATGCCCTGGTTTTGGTTGAA
mIL22-F	ATGAGTTTTTCCCTTATGGGGAC
mIL22-R	GCTGGAAGTTGGACACCTCAA
mIL23-F	GTGACCCACAAGGACTCAAGGA
mIL23-R	AGCAGGCTCCCCTTTGAAGAT
mIL12b-F	CTGGAGCACTCCCCATTCCTA
mIL12b-R	GCAGACATTCCCGCCTTTG
mCD38-F	TCTCTAGGAAAGCCCAGATCG
mCD38-R	GTCCACACCAGGAGTGAGC
mPtgs2-F	TGAGCAACTATTCCAAACCAGC
mPtgs2-R	GCACGTAGTCTTCGATCACTATC
miNos-F	GTTCTCAGCCCAACAATACAAGA
miNos-R	GTGGACGGGTCGATGTCAC
mIL1β-F	GCAACTGTTCCTGAACTCAACT
mIL1β-R	ATCTTTTGGGGTCCGTCAACT
mCD206-F	CTCTGTTCAGCTATTGGACGC
mCD206-R	CGGAATTTCTGGGATTCAGCTTC
mMgl2-F	TTAGCCAATGTGCTTAGCTGG
mMgl2-R	GGCCTCCAATTCTTGAAACCT
mYm1-F	CAGGTCTGGCAATTCTTCTGAA
mYm1-R	GTCTTGCTCATGTGTGTAAGTGA
mArg1-F	CTCCAAGCCAAAGTCCTTAGAG
mArg1-R	AGGAGCTGTCATTAGGGACATC
mIL10-F	GCTCTTACTGACTGGCATGAG
mIL10-R	CGCAGCTCTAGGAGCATGTG
mPPARγ-F	TTTTCAAGGGTGCCAGTTTC
mPPARγ-R	AATCCTTGGCCCTCTGAGAT
mCCND1-F	GCGTACCCTGACACCAATCTC
mCCND1-R	CTCCTCTTCGCACTTCTGCTC
mEgr2-F	GCCAAGGCCGTAGACAAAATC
mEgr2-R	CCACTCCGTTCATCTGGTCA
mAdgre1-F	TGACTCACCTTGTGGTCCTAA
mAdgre1-R	CTTCCCAGAATCCAGTCTTTCC
mH2ab1-F	GCGAAGAAGACGGAAAGTGAC
mH2ab1-R	GCGAGGAATACAGGAGCAGAA
mIL23r-F	TTCAGATGGGCATGAATGTTTCT
mIL23r-R	CCAAATCCGAGCTGTTGTTCTAT
mCxcl2-F	CCTCAACGGAAGAACCAAAGAG
mCxcl2-R	CTCAGACAGCGAGGCACATC
mCxcl16-F	CCTTGTCTCTTGCGTTCTTCC
mCxcl16-R	TCCAAAGTACCCTGCGGTATC
mCx3cl1-F	ACGAAATGCGAAATCATGTGC

mCx3cl1-R	CTGTGTCGTCTCCAGGACAA
mCsf1r-F	CTTGTCTGGCCAGCAATGATGTTG
mCsf1r-R	AGGCTCTGCTCAGAGGTCAAGTTT
mCx3cr1-F	GAGTATGACGATTCTGCTGAGG
mCx3cr1-R	CAGACCGAACGTGAAGACGAG

Supplementary Table 2. Antibody information list.

Myeloid cell panel

Antibodies	Color	Company	Cat	Clone	Host	Comments
Zombie UV	BUV395	Biolegend	423107		rat	Stain dead cells
CD45.2	Alexa 700	Biolegend	109822	104	mouse	Surface
MHC II (I-A/I-E)	APC-Cy7	Biolegend	107628	M5/114.15.2	mouse	Surface
CD64	APC (AF647)	Biolegend	139306	X54-4/7.1	mouse	Surface
F4/80	BV421	Biolegend	123131	BM8	rat	Surface
CD11b	BV510	Biolegend	101245	M1/70	rat	Surface
Ly6C	BV605	Biolegend	128035	HK1.4	mouse	Surface
CD11c	BV785	Biolegend	117335	N418	mouse	Surface
CX3CR1	PE	Biolegend	149006	SA011F11	mouse	Intracellular
Ly6G	PE-Cy7	Biolegend	127618	1A8	mouse	Surface
CD206	PerCP-Cy5.5	Biolegend	141715	C068C2	rat	Surface
CD68	FITC(AF488)	Biolegend	137005	FA-11	rat	Surface
IL-23 p19	APC (AF647)	BD Bioscience	565317	71-1183 (RU(	mouse	Intra-cellular

T cell panel

Antibodies	Color	Company	Cat	Clone	Host	Comments
Zombie UV	BUV395	Biolegend	423107		rat	Stain dead cells
CD3e	APC-Cy7	Biolegend	100330	145-2C11	hamster	surface
CD45.2	AF700	Biolegend	109822	104	mouse	surface
CD8a	BV605	Biolegend	100743	53-6.7	rat	surface
CD4	BV510	Biolegend	100553	RM4-5	rat	Surface
TCRβ	PE	Life Technolog	12-5961-82	H57-597	hamster	Surface
RORγt	BV421	BD Horizon	BDB562894	Q31-378	mouse	Intra-cellular
IL17A	PE-Cy7	Biolegend	506921	TC11-18H10	rat	Intra-cellular
IFNγ	PerCP-Cy5.5	Biolegend	505821	XMG1.2	rat	Intra-cellular

Supplementary Table 3. Human CAC tissues information source data list.

Tissues	Enrolled datasets
Normal controls,<br>N (n = 24)	GSM2332098, GSM2332100, GSM2332101, GSM2332102, GSM2332103, GSM2332104, GSM2332105, GSM2332107, GSM2332114, GSM2332115, GSM2332116, GSM2332117, GSM2429455, GSM2429457, GSM2429458, GSM2429459, GSM2429460, GSM2429461, GSM2429462, GSM2429464, GSM2429471, GSM2429472, GSM2429473, GSM2429474
Ulcerative colitis with neoplasia,<br>UCN (n = 11)	GSM915460, GSM915461, GSM915462, GSM915463, GSM915464, GSM915465, GSM915466, GSM915467, GSM915468, GSM915469, GSM915470

Abbreviations used in this paper: Abbreviations used in this paper: CRC, colorectal cancer; CAC, colitis-associated cancer; IBD, inflammatory bowel diseases; BA, bile acids; FXR, Farnesoid X receptor; AOM, azoxymethane; DSS, dextran sodium sulfate; WT, wild-type; IL, interleukin; TNFα, tumor necrosis factor–a; IFNγ, interferon gamma; LPS, lipopolysaccharide; mRNA, messenger RNA; IHC, immunohistochemical; LP, lamina Propria; IEL, Intraepithelial lymphocytes; ILC, innate lymphoid cell; ISC, intestinal stem cell; FexD, Fexaramine D; OCA, Obeticholic acid; DCA, deoxycholic acid; T-β-MCA, tauro-beta-muricholic acid; LCA, Lithocholic acid; BMDMs, Bone Marrow-Derived Macrophages; GEO, Gene Expression Omnibus; TCGA, The Cancer Genome Atlas.
